# The Gestational Effects of Maternal Bone Marker Molecules on Fetal Growth, Metabolism and Long-Term Metabolic Health: A Systematic Review

**DOI:** 10.3390/ijms23158328

**Published:** 2022-07-28

**Authors:** Angelos Dimas, Anastasia Politi, Alexandra Bargiota, Theodoros Panoskaltsis, Nikolaos F. Vlahos, Georgios Valsamakis

**Affiliations:** 1 3rd University Department of Obstetrics & Gynecology, Attikon University Hospital, Medical School of Athens, Ethnikon and Kapodistriakon University of Athens, 12462 Athens, Greece; 2 Obst & Gynae Department, University Hospital of Ioannina, Stavros Niarchos Ave., 45500 Ioannina, Greece; 3 Nephrology Department, University Hospital of Ioannina, Stavros Niarchos Ave., 45500 Ioannina, Greece; anastasia.k.politi@gmail.com; 4 Department of Endocrinology and Metabolic Diseases, Medical School, Larissa University Hospital, University of Thessaly, 41334 Larissa, Greece; abargio@yahoo.gr; 5 2nd University Department of Obstetrics & Gynecology, “Aretaieion” University Hospital, Medical School of Athens, Ethnikon and Kapodistriakon University of Athens, 12462 Athens, Greece; panoskaltsistheo@gmail.com (T.P.); nfvlahos@gmail.com (N.F.V.); 6 Endocrine Unit, 2nd Department of Obstetrics and Gynecology, National and Kapodistrian University of Athens, “Aretaieion” University Hospital, 11528 Athens, Greece

**Keywords:** vitamin D, sclerostin, osteocalcin, sRANKL, bone metabolism, fetal growth, fetal metabolism, birthweight, endocrine health, offspring metabolism

## Abstract

Fetal exposure in adverse environmental factors during intrauterine life can lead to various biological adjustments, affecting not only in utero development of the conceptus, but also its later metabolic and endocrine wellbeing. During human gestation, maternal bone turnover increases, as reflected by molecules involved in bone metabolism, such as vitamin D, osteocalcin, sclerostin, sRANKL, and osteoprotegerin; however, recent studies support their emerging role in endocrine functions and glucose homeostasis regulation. Herein, we sought to systematically review current knowledge on the effects of aforementioned maternal bone biomarkers during pregnancy on fetal intrauterine growth and metabolism, neonatal anthropometric measures at birth, as well as on future endocrine and metabolic wellbeing of the offspring. A growing body of literature converges on the view that maternal bone turnover is likely implicated in fetal growth, and at least to some extent, in neonatal and childhood body composition and metabolic wellbeing. Maternal sclerostin and sRANKL are positively linked with fetal abdominal circumference and subcutaneous fat deposition, contributing to greater birthweights. Vitamin D deficiency correlates with lower birthweights, while research is still needed on intrauterine fetal metabolism, as well as on vitamin D dosing supplementation during pregnancy, to diminish the risks of low birthweight or SGA neonates in high-risk populations.

## 1. Introduction

In recent years, it has been well-established that intrauterine life is a particularly vulnerable period of development, characterized by plasticity. Exposure to adverse environmental factors can interact with genotypic variation, affecting profoundly the ability of the organism to cope effectively with its environment in later life [1,2]. These factors include maternal nutritional patterns, diseases, drug use or exposure to chemicals, maternal microbiome, and several maternal or fetal stressors. For example, offspring of pregnancies during the Dutch famine suffered from adverse metabolic phenotypes and intrauterine growth retardation leading to lower birthweights and decreased head circumferences [3,4]. Fetal gender, as well as the type and exact developmental period of exposure, also seem to play a profound role in this process of fetal developmental programming [2].

During normal human gestation, maternal bone turnover—as reflected by various biochemical serum molecules—increases, and a state of a reversable bone mineral density and bone mass reduction develops [5]. One of these biomarkers can be considered vitamin D (or calciferol). Except for its pivotal role in maternal calcium homeostasis regulation and bone mineralization, Vit-D is involved in immunological, circulatory, neurological, and biological process modulation [6,7,8,9]. Maternal Vit-D also regulates endometrium receptivity and embryogenesis, while its levels are crucial for trophoblast invasion, fetal skeletal development, as well as fetal growth [9,10,11,12]. Furthermore, numerous studies suggest that maternal Vit-D status is related with adverse pregnancy outcomes, such as preeclampsia, gestational diabetes mellitus (GDM), low birth weight, prematurity, impaired offspring neurodevelopment, neonatal body composition, as well as newborn long-term metabolic health [9,13,14,15,16].

Other maternal molecules involved in bone metabolism include sclerostin, osteocalcin, osteoprotegerin, and receptor activator of nuclear factor-κB ligand (RANKL). Osteocalcin serves as a marker of bone formation. However, recent studies revealed its emerging endocrine function as an energy and glucose homeostasis regulator [17,18]. RANKL is produced by osteoblasts and activated T-lymphocytes, promoting osteoclast differentiation, activation, and survival. Its binding with osteoclast transmembrane receptor RANK results in bone resorption and calcium mobilization from bone tissue [19,20,21]. Osteoprotegerin is a TNF-like protein secreted by osteoclasts, which inhibits bone resorption and osteoclast formation, hindering the binding between RANKL and RANK, thus acting as a soluble decoy receptor for RANKL [21,22]. Sclerostin is expressed in mature osteocytes and constitutes a potent bone formation inhibitor, while stimulating bone resorption by blocking the physiological Wnt signaling pathway and downregulating osteoprotegerin expression [23]. Nevertheless, several reports implicate sclerostin in B-cells apoptosis, the occurrence of vascular calcifications, as well as adipocyte differentiation and fat production modulation [23,24,25,26,27].

There is increasing evidence supporting the emerging interaction between bone and glucose metabolism [17,28]; however, these mechanisms are not yet fully elucidated during normal human pregnancy. The aim of this study is to systematically analyze the literature and summarize the possible effects of maternal bone turnover molecules on fetal growth and metabolism, birth anthropometrics, and metabolic health of offspring in later life.

## 2. Methods

Our aim was to explore the effects of maternal bone metabolism molecules during pregnancy, on intrauterine fetal growth (including ultrasound-derived measures, such as estimated fetal weight, femur length, tibia length, head or abdominal circumference, and subcutaneous fat deposition), fetal metabolism, anthropometric measurements at birth (including birth weight, abdominal, body composition, head, and waist circumference at birth), and the longer-term metabolic wellbeing of offspring. We used the medical search engines Scopus and PubMed. Our search was performed using the following keywords, separately or in combination: maternal bone molecules, maternal bone markers, maternal bone biomarkers, maternal bone metabolism, sclerostin, sRANKL, soluble receptor activator of nuclear factor-κB ligand, osteocalcin, osteoprotegerin, 25-hydroxyvitamin D3, cholecalciferol, 25(OH)D, Vitamin D, fetal growth, fetal metabolism, birth anthropometry, birth weight, offspring metabolism. We sought human, prospective, and retrospective cohort studies, systematic reviews, and meta-analyses in English language, published in medical journals prior to 1 May 2022.

**Figure 1:**
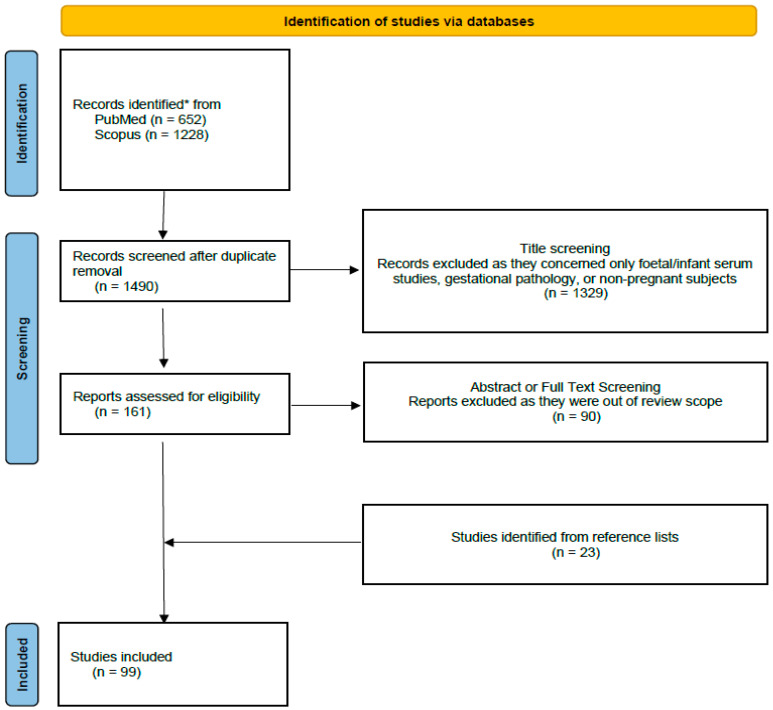
Study flowchart [29] *(((“maternal bone molecules” OR “maternal bone markers” OR “maternal bone biomarkers” OR “maternal bone metaboli*” OR sclerostin OR sRANKL OR “soluble receptor activator of nuclear factor-κB ligand” OR osteocalcin OR osteoprotegerin OR “25-hydroxyvitamin D3” OR “cholecalciferol” OR “Vitamin D”) AND (“fetal growth” OR “fetal metaboli*” OR “birth anthropometr*” OR “birth weight” OR “offspring metaboli*”))).

Our search detected initially 1880 published papers. After duplicates removal, 1490 studies underwent the title screening phase; 1329 studies were excluded during this process because they were out of the review scope, as they either concerned only infant or fetal and not maternal serum bone molecules, non-pregnant subjects, or referred to gestational pathology, such as gestational diabetes mellitus or preeclampsia. The remaining 161 full-text papers were assessed for eligibility. Finally, 71 studies were selected that focused on maternal bone turnover biomarkers influencing fetal growth, fetal and newborn metabolism, birth anthropometry, or the metabolic health of offspring in healthy pregnancies. Reference lists of relevant studies were screened for studies not detected by our initial search. Finally, 94 studies were included in our systematic review. The flow chart of the process is depicted in Figure 1.

## 3. Results and Discussion

### 3.1. Maternal Bone Turnover Molecules during Pregnancy and Intrauterine Fetal Growth

Of the known bone markers, Vit-D levels assessed by maternal serum 25-hydroxyvitamin-D or 25(OH)D, is the most extensively studied. Nevertheless, 25(OH)D has its limitations, as several studies underline the various confounding factors affecting its serum levels, namely skin pigmentation, maternal age, gestational age, parity, adiposity status, ethnicity, geographical residence, season and pregnancy trimester at sampling, maternal sun exposure, diet, and Vit-D supplementation [30,31,32,33,34,35]. Additionally, differences in methods to assess Vit-D levels, as well as lack of consensus regarding its cutoffs, all may have contributed to discordant study results in the literature. Published studies assessing the potential interactions between maternal bone turnover molecules and intrauterine fetal growth are summarized in Supplemental Table S1.

#### 3.1.1. Maternal Vit-D during Early Gestation and Intrauterine Fetal Growth

Several studies assessed maternal levels of Vit-D during early pregnancy and its implications in fetal in utero growth and development. In a large retrospective cohort study of 15,651 pregnancies in China by Zhang et al. [36], fetuses of Vit-D insufficient/deficient mothers during early pregnancy had a decreased crown–rump length, compared to the Vit-D sufficient group. In the same maternal group, the risk of early fetal growth restriction was also increased by 13%. The same study reported a significant, combined effect of maternal overweight or obesity and maternal Vit-D concentrations on fetal crown–rump length and the risk of fetal intrauterine growth restriction. Researchers concluded that sufficient maternal serum 25(OH)D levels during the first trimester of gestation was a protective factor for early fetal growth restriction, especially in the presence of maternal overweight or obesity. Similarly, Judistiani et al. [37], in their prospective study of 203 pregnancies in Indonesia, found significant positive associations between first trimester maternal Vit-D levels with third trimester fetal biparietal diameter and abdominal circumference, even after adjustment for maternal age, pre-pregnancy BMI and parity. In line with forementioned results, Walsh et al. [38] demonstrated that seasonal variation of 25(OH)D in maternal serum has detrimental effects on fetal development. They prospectively analyzed 60 pregnancies in Ireland, categorized in two subgroups, a winter and a summer cohort, recruited in early pregnancy during September/October and during March/April, respectively. In the winter cohort, early pregnancy 25(OH)D maternal levels were positively correlated with fetal femur length at 20 weeks, while maternal serum 25(OH)D concentrations at 28 weeks correlated positively with femur length at 34 weeks of gestation. Furthermore, neonatal length at birth was significantly increased in women with Vit-D levels above the median concentration at early gestation.

On the other hand, in a Korean study, Lee et al. [39]—studying prospectively 245 pregnancies in all three trimesters—found a negative correlation between first trimester maternal 25(OH)D level and growth velocity of fetal biparietal diameter between 20–22 and 32–34 gestational weeks. Contrarily, a positive correlation between the difference of maternal 25(OH)D levels between 12–14 and 20–22 weeks and growth velocity of fetal biparietal diameter between 20–22 and 32–34 weeks was observed. Authors concluded that the changes of 25(OH)D levels between 12–14 and 20–22 weeks seem to influence fetal biparietal diameter growth, while they did not affect the growth of other fetal parameters studied, such as head and abdominal circumference or femur and humerus length.

Lastly, Aydeniz et al. [40], studying retrospectively 154 maternal–fetal dyads in Turkey between 12 to 14 weeks of gestation, did not find any significant associations between second trimester fetal femur length and first trimester maternal serum 25-hydroxyvitamin-D3. Similar conclusions were reached in the prospective study by Morales et al. [41], as well as the cross-sectional study by Fernández-Alonso et al. [42], both conducted in Spain. The authors of the first study reported no association of maternal 25(OH)D3 levels with fetal femur length, while a weak inverse relationship with biparietal diameter at 34 weeks of gestation was noted. However, maternal 25(OH)D3 deficit was associated with increased risk of fetal overweight as assessed by ultrasound (either AC or estimated fetal weight ≥ 90th centile). Fernández-Alonso et al., on the other hand, demonstrated that maternal 25(OH)D levels during first trimester do not correlate, neither with fetal crown–rump length nor with nuchal translucency in 498 healthy maternal–fetal dyads studied.

Interpretation of the above study results can be difficult due to significant heterogeneity in sample sizes and methods used, hindering direct comparisons. For example, the studies of Aydeniz, Judistiani, Lee and Walsh et al. al seem to be too underpowered to extract robust associations. The work of Zhang et al., although with a large sample, is a retrospective one, bearing all the inherent weaknesses of retrospectivity. Morales et al., on the other hand, measured 25(OH)D_3_ levels in maternal serum, in contrast with the majority of other studies that measured maternal 25(OH)D levels. Furthermore, most of the studies examined populations of ethnicities and used different 25(OH)D cutoffs, or used its levels either as a categorical or continuous variable, further complicating efforts to pool study samples for metanalysis. Lastly, only the study of Judistiani et al. adjusted data for covariates known to impact fetal growth and/or maternal Vit-D status.

#### 3.1.2. Maternal Vit-D during Second and Third Trimesters and Intrauterine Fetal Growth

Perhaps maternal Vit-D status during second and third trimesters may influence intrauterine growth more profoundly. A large population-based prospective cohort study from the Netherlands [43] in a multiethnic population assessed maternal 25(OH)D levels during the second and third trimesters of pregnancy in relation to intrauterine fetal growth. Low maternal 25(OH)D in second trimester was associated with restricted fetal head circumference growth from the second trimester and until birth. Similar associations were reported for fetal body length and weight growth. Moreover, lower Vit-D concentrations correlated with neonates with low birthweights and small size for gestational age (SGA). However, when analysis was restricted to Europeans only, associations attenuated for some of the outcomes. Similarly, in their study Sarma et al. [44], assessing maternal Vit-D levels of 250 pregnancies during third trimester in India, found that fetal femur length at 34 weeks, as well as birth length, were significantly shorter in mothers with low Vit-D serum levels, while no significant differences were found regarding fetal birth weight and head circumference.

Young et al., studying prospectively 171 healthy adolescent pregnancies in USA, demonstrated that adequate mid-gestation maternal 25(OH)D levels are positively associated with fetal femur and humerus z-scores, even after adjustment for covariates. Interestingly enough, this association was evident only when maternal calcium intake was lower than 1050 mg per day. Another prospective UK cohort study [45] with 424 participants recruited in the Southampton Women Survey did not find significant correlations between maternal 25(OH)D levels at 34 weeks and fetal femur length, but lower maternal Vit-D concentrations were associated with greater femoral metaphyseal cross-sectional area and a higher femoral splaying index (femur length / distal metaphyseal cross-sectional area), both at 19 and at 34 weeks of pregnancy. Authors concluded that maternal Vit-D insufficiency may influence fetal femoral development early in pregnancy. Moreover, Ioannou et al. [46], studying another sub-cohort of 357 pregnancies from the same survey, demonstrated that maternal 25(OH)D concentrations at 34 weeks correlate significantly with fetal femoral volume and proximal metaphyseal diameter. Findings suggest that anabolic effects of maternal Vit-D may be more evident in fetal bone girth than length. Nevertheless, the effect of maternal Vit-D levels on femoral volume was attenuated on multiple regression model.

Another prospective cohort study of 10.913 pregnancies by Liu et al., investigated the combined effects of maternal Vit-D deficiency at 24–28 weeks of pregnancy and gestational diabetes mellitus (GDM) on fetal growth trajectories in China. They concluded that both conditions were independently associated with an increased risk of excessive fetal growth, as assessed by estimated fetal growth z-score. A similar positive correlation was observed by Lee et al. [39], between the difference of maternal 25(OH)D levels between 12 to 14 and 20 to 22 weeks and growth velocity of fetal biparietal diameter between 20 to 22 and 32 to 34 weeks of gestation. In contrast, Marçal et al. [47], in their cross-sectional study of 87 gestations in Brazil, reported that maternal serum Vit-D levels at 26 to 36 weeks did not differ significantly among mothers of appropriate for gestational age, small for gestational age, and growth-restricted neonates, according to the sonographic estimated fetal weight.

In our search, we managed to identify only one systematic review of observational studies assessing fetal biometry by ultrasound, in relation to maternal Vit-D serum levels. Authors concluded that low maternal 25(OH)D concentrations seem to affect fetal bone growth, especially with concomitant insufficient calcium intake [48].

In this section of our systematic review, clear conclusions are difficult to draw. Firstly, while the studies of Ioannou and Mahon et al. reported associations between maternal Vit-D status and fetal femoral development, the emerging sonographic indices measured are not widely used and are difficult to be replicated by non-experts, and comparisons are therefore impossible to date. Nevertheless, although the two sub-studies derived from the same cohort, authors used different Vit-D cutoffs to draw conclusions. Moreover, the large prospective study by Liu et al. seem to oppose the results by others, while in both, the lack of adjustment for covariates and the inclusion of gestational diabetes mellitus cases may have tampered with results. Lack of data correction for covariables in all other studies reviewed in this section—apart from the research of Young and Miliku et al.—combined with the inaccurate nature of sonographic measurements, with great inter- and intra-observer variability, make their interpretation a difficult task.

#### 3.1.3. Maternal Sclerostin and sRANKL during Pregnancy and Intrauterine Fetal Growth

Regarding other maternal bone turnover molecules, studies in the literature are scarce. In their recent study of 100 healthy pregnancies, Mastorakos et al. [49] demonstrated that both maternal sclerostin, as well as maternal sRANKL levels, had a positive correlation with fetal abdominal circumference during the second trimester of pregnancy. Moreover, maternal sclerostin was also positively correlated with fetal birthweight, while maternal sRANKL with fetal subcutaneous fat thickness and sagittal abdominal diameter, as assessed by sonography. In fact, mothers with greater than median serum concentrations of sRANKL during the second trimester carried fetuses with greater AC and sagittal abdominal diameter measurements, as well as fetal subcutaneous abdominal thickness, than fetuses of mothers with lower sRANKL levels. Third trimester maternal sclerostin and sRANKL levels were also correlated with fetal sagittal abdominal diameter. Maternal sRANKL was additionally positively correlated with fetal sagittal abdominal diameter during late pregnancy. The study concluded that maternal second trimester serum sclerostin levels were the best positive predictors of birth weight, even after adjustment for gestational age, maternal BMI and insulin concentrations, fetal sex and other covariables.

The emerging role of sclerostin as a mediator of adipose tissue hypertrophy and differentiation has been already demonstrated in rodents [50,51], whereas sRANKL seems to be implicated in glucose metabolism regulation through insulin sensitivity regulation in animal studies [52,53]. Thus, maternal bone metabolism biomarkers may not be implicated only in fetal bone growth, but also indirectly in adipose tissue accumulation and deposition.

### 3.2. Maternal Bone Turnover Molecules during Pregnancy and Anthropometrics at Birth

Neonatal birth weight and anthropometrics at birth in general have been widely used to assess fetal growth. Nevertheless, neonatal birth weight alone, as a method of fetal growth assessment, has its limitations, due to different fetal growth patterns and body compositions leading to similar birthweights, and therefore, other anthropometric indices should be considered as well.

#### 3.2.1. Maternal Vit-D during Early Gestation and Anthropometrics at Birth

In the international literature, several studies can be found assessing maternal Vit-D status during pregnancy and its effects on anthropometric measurements at birth. Their main findings are summarized in Supplemental Table S2. Many of them have reported a relation of maternal Vit-D levels with neonatal anthropometric indices at birth. A Chinese prospective study of 747 pregnancies found a non-linear, positive relation between maternal 25(OH)D and neonatal birthweight, as well as head circumference at birth. Per 1 ng/mL increase in maternal 25(OH)D (and up to 20 ng/mL), birth weight and head circumference increased by 69 gr and 0.31 cm, respectively. Even after covariates adjustment, the risk of SGA increased by 9% for each one unit decrease of maternal plasma 25(OH)D concentration [54]. Similarly, a retrospective Australian study of 304 pregnancies showed that increased maternal total and free 25(OH)D levels were associated with higher neonatal birthweight, when data are adjusted for maternal age, BMI, and ethnicity [55]. Another Australian prospective cohort study including 402 women concluded that maternal serum 25(OH)D levels at recruitment (<16 weeks) in smokers were inversely related with offspring fat mass percentage, but positively related with neonatal lean mass. No association was evident at 28–32 weeks or in non-smokers [16].

In line with previous results were studies by Leffelaar et al. [56] and van den Berg et al. [57] from the Netherlands. They demonstrated that first trimester Vit-D deficient mothers have a higher risk of giving birth to an SGA neonate. Interestingly enough, van den Berg et al. underlined that maternal Vit-D levels seem to be a modifiable contributor to SGA neonates, especially for overweight mothers and those who conceived during the winter period. Similarly, Leffelaar et al. reported infants with lower birthweights born to Vit-D-deficient mothers, compared with women with adequate Vit-D concentrations [56]. Moreover, a large multicentric retrospective analysis measuring maternal 25(OH)D serum levels at booking and every 8 weeks thereafter reported that mothers with Vit-D levels of 37.5 nmol/L or higher gave birth to neonates 46 g heavier and with 0.13 cm larger head circumferences, compared to mothers with lower than 37.5 nmol/L Vit-D levels. Researchers concluded that maternal 25(OH)D levels of 37.5 nmol/L or higher versus less than 37.5 nmol/L during first trimester—but not during second trimester—are associated with half the risk of a small-for-gestational-age newborn [58].

In contrast, the prospective cohort study of Rodriguez et al. [59] in Spain showed a negative correlation of first trimester maternal Vit-D levels with neonatal head circumference at birth. The same study did not report any further associations of maternal Vit-D levels neither with GDM, preterm delivery, fetal growth restriction, SGA, or other birth anthropometric outcomes. However, several other studies did not find any associations at all regarding early pregnancy Vit-D levels and neonatal birth anthropometrics at birth. A multiethnic, prospective cohort study of 5109 Australian pregnancies failed to correlate first trimester low maternal 25(OH)D levels with adverse neonatal outcomes [60]. Similar results were reported by two small prospective studies in Indonesia [61,62]. Lastly, the prospective study of Park et al. [63] in Korea concluded that birth weight is independent of maternal serum 25(OH)D levels during all trimesters of pregnancy.

#### 3.2.2. Maternal Vit-D during Mid—Gestation and Anthropometrics at Birth

The majority of studies addressing maternal Vit-D levels in relation to neonatal birth weight and anthropometrics at birth have been conducted during the second trimester of pregnancy. Most of them reported significant correlations between maternal Vit-D levels and neonatal anthropometric indices at birth. A large retrospective study of 15,724 pregnancies in China reported a decreased risk of a large-for-gestational-age (LGA) neonate in mothers with severe 25(OH)D deficiency [64]. However, they noted that the simultaneous presence of maternal Vit-D deficiency and GDM could in fact increase the risk of LGA. Another retrospective study of 2814 Chinese pregnancies concluded also that higher early pregnancy Vit-D levels are associated with lower risk of low-birthweight neonates, after adjusting for co-variates [65]. In Ireland, Casey et al. [66], investigated a multiethnic, 1585-women sub-cohort of HAPO study. They reported that, after adjusting for cofounders, the doubling of maternal 25(OH)D levels at 28 weeks positively affected both newborn birth weight and birth length by 0.05 and 0.07 standard deviation scores, respectively. Moreover, serum 25(OH)D concentrations of 890 participants of the Western Australian Pregnancy Cohort (Raine) Study were evaluated at 18 weeks of gestation by Mosavat et al. [67] They found a positive association between maternal 25(OH)D levels with neonatal birth weight, body length, and head circumference. They also demonstrated that low maternal Vit-D levels are associated with GDM development, although maternal ethnicity attenuated this relation. The previous study validated the work of Morley et al. [68]. Studying prospectively 374 Australian pregnancies, they measured maternal 25(OH)D levels at 16 and 28 weeks of gestation. The study concluded that low maternal Vit-D levels at 28–32 weeks are associated with lower mean knee-to-heel length and reduced mid-upper arm and calf circumferences, compared to Vit-D-sufficient mothers. However, maternal 25(OH)D levels in early pregnancy were not associated with any of the studied birth parameters.

Four multiethnic studies from USA reached to similar conclusions. In their prospective study of 792 pregnancies, Gernand et al. [69] concluded that maternal Vit-D status during second trimester is inversely associated with risk of an SGA neonate in white, non-obese women. However, the sample consisted of high risk for preeclampsia development participants. Burris et al. [70], prospectively studying 1067 white and 236 black pregnant women, found that the odds of an SGA neonate were significantly higher when maternal 25(OH)D levels were lower than 25 nmol/L. They also suggested that Vit-D status may be implicated in racial disparities found in SGA births among black and white women. Similarly, the retrospective cohort study of 2473 participants by Eckhardt et al. [71] reported that infants of mothers with 25(OH)D ≥ 30 nmol/L had higher weight and BMI z-scores at birth, compared to mothers with 25(OH)D ≤ 30 nmol/L. Furthermore, Tian et al. [72] in their study evaluated maternal 25(OH)D levels from 4 to 29 weeks of gestation in 2558 pregnancies. They reported a positive association between early and mid-pregnancy maternal 25(OH)D concentrations with birthweight for gestational age among non-Hispanic black male and female neonates, as well as non-Hispanic white male infants.

Lastly, Eggemoen et al. [73] investigated possible associations between maternal 25(OH)D levels at 15 to 28 weeks in 719 pregnancies in Norway and neonatal birth anthropometrics. They reported a strong positive association with birth weight when data adjusted for maternal age, parity, educational level, pre-pregnancy BMI, season gestational age, and neonate sex. However, this correlation attenuated after additional adjustment for ethnicity. Similarly, maternal 25(OH)D levels were positively related with crown–heel length, head circumference, abdominal circumference, and ponderal index at birth, both at 15 and 28 weeks, while the associations lost significance after ethnicity adjustment. Authors also reported a positive, sex-dependent effect of maternal 25(OH)D levels on abdominal circumference in female neonates and a negative effect on skinfolds sum in males in the ethnicity adjusted model [73]. On the contrary, Wen et al. [74] in their nested case control study reported a 33% increased risk of macrosomia in Chinese women with Vit-D levels <50 nmol/L compared to women with levels from 50 to 74.9 nmol/L.

Assessing maternal 25(OH)D status in relation to offspring’s adiposity, Tint et al. [75] reached to similar conclusions. Studying 292 Asian mothers and their neonates at 26–28 weeks of pregnancy, they observed an inverse correlation of maternal 25(OH)D concentrations with both neonatal superficial and deep subcutaneous abdominal adipose tissue, a relation that persisted even after data adjustment for maternal glucose levels during pregnancy. As a result, neonates of mothers with Vit-D inadequacy had a greater abdominal subcutaneous tissue volume, compared to Vit-D-sufficient mothers. Contrarily, in their prospective study of 202 pregnancies in Norway, Godang et al. [76] found a strong positive association between maternal and fetal umbilical cord plasma 25(OH)D concentrations. In turn, fetal umbilical cord plasma 25(OH)D levels were positively associated with neonatal total body fat mass at birth. Nevertheless, maternal levels per se were not significantly associated with offspring’s body fat mass, while a seasonal fluctuation of maternal and neonatal 25(OH)D levels at northern latitudes was noted.

Bodnar et al. [77], studying a sub-cohort of a large prospective study with 412 participants, found a U-shaped relation between maternal serum 25(OH)D and risk of an SGA neonate in white mothers, while there was no relation between maternal Vit-D levels and SGA risk among black mothers. Researchers also reported one single nucleotide polymorphism (SNP) in white women and three SNPs in black women belonging to the Vit-D receptor gene (VDR), which were significantly associated with birth of a small-for-gestational-age neonate. Moreover, a recent large retrospective cohort study from southern China with 10.586 participants did not find significant differences in maternal Vit-D levels between the three neonatal groups (small, appropriate, and large for gestational age). The authors deduced that maternal 25(OH)D levels was not an effective predictor, neither of SGA or LGA neonates [78]. In line with this study, in their large prospective studies, both Boyle et al. [79] in New Zealand, as well as Zhou et al. [80] in southern China, reported no significant associations between midgestational maternal levels of Vit-D and adverse neonatal outcomes including SGA neonates.

#### 3.2.3. Maternal Vit-D during Third Trimester or Peripartum and Anthropometrics at Birth

A few researchers sought to investigate the possible relation of maternal Vit-D levels in late gestation with neonatal birth anthropometric measurements. A Swedish prospective cohort of 1816 women demonstrated that maternal levels of 25(OH)D ≥40 ng/mL were associated with lower odds of an SGA or low birthweight neonate, in comparison to 25(OH)D deficient mothers. In fact, the lowest risk of SGA presented in those mothers with a 25(OH)D level rise of 30 ng/mL or more between the first and third trimesters. The same study did not find any associations between first-trimester maternal 25(OH)D status with neonatal birth anthropometrics [81]. Similarly, a significantly higher neonatal birth weight, birth length, and head circumference was reported in pregnancies in the 25(OH)D sufficient group compared to deficient mothers was reported in a small Iranian cross-sectional study of 88 participants [82]. Contrarily, two small prospective studies from Slovenia and Poland reported no significant associations between maternal 25(OH)D levels and newborn anthropometric indices [83,84].

Other studies sought to evaluate peripartum maternal Vit-D status. Wang et al. [85] studied 1978 pregnancies in China and concluded that maternal Vit-D deficiency increased the risk of a low birthweight or SGA neonate, in line with two other small studies from India [86] and Iraq [87]. Authors also stated that Vit-D deficiency independently increased the risk of GDM development, while Vit-D supplementation during pregnancy reduced the risk of low birthweight neonates.

In contrast, Lee et al. [88], in their prospective study of 575 pregnancies, reported no significant associations of maternal Vit-D levels with intrauterine growth restriction or other adverse neonatal outcomes, such as low birth weight, in agreement with a smaller prospective study of 172 participants in Seychelles [89]. No difference in neonatal weight, height, or head circumference was also reported by Sandal et al. between newborns of mothers with Vit-D deficiency and those with sufficient Vit-D levels in the first 24 h after parturition [90]. However, data should be carefully evaluated, as the primary outcome of this study was different. In Belgium, Dullaert et al. [91] found a significant association between maternal Vit-D levels lower than 10 ng/mL and neonatal birthweight lower than the 10th centile in the crude model, but the association did not reach statistical significance in multivariate analysis. Similar results were reported recently in a small cross-sectional study of 106 pregnancies in Iran [92].

#### 3.2.4. Maternal Vit-D throughout Gestation and Anthropometrics at Birth

Except for the above studies, which sought to evaluate maternal Vit-D levels in specific timepoints during pregnancy, others assessed levels longitudinally throughout gestation. A longitudinal prospective study by Francis et al. [93] measured maternal 25(OH)D serum levels at different timepoints during pregnancy and stratified data for pre-pregnancy maternal BMI. In overweight or obese mothers, maternal 25(OH)D levels below 50 nmol/L at 10–14 weeks correlated with lower neonatal birth length and birthweight z-score, whereas 23–31 gestational weeks correlated with lower birth length and number of skinfolds. Regarding normal-weight women, maternal levels lower than 50 nmol/L at 10–14 gestational weeks were associated with lower neonatal sum of skinfolds, but with larger birthweight z-scores at 23–31 weeks and with both higher birthweight z-score and birth length at 33–39 weeks of pregnancy. Boghossian et al. prospectively recruited 252 pregnancies in USA and evaluated maternal 25(OH)D levels between Caucasian and African-American women at 11–21, 20–25, and 30–36 gestational weeks. Newborns of Vit-D-deficient mothers had lower neonatal bone mineral density, bone mineral content, total fat mass, lean mass, and birthweight compared to newborns of Vit-D-sufficient mothers in the crude model. However, after adjusting for covariates, only the association for bone mineral density remained significant. Neonatal lean mass and birthweight were significantly correlated with maternal 25(OH)D deficiency among males in the neonatal sex-adjusted model [94]. In a Brazilian cohort of 168 participants, a direct association was reported between the mean rate of increase in 25(OH)D maternal serum levels during pregnancy and birthweight z-score, as well as a similar tendency for birth length z-score. Moreover, maternal Vit-D levels were positively associated with an increased risk of an LGA neonate [95].

Finally, some other studies measured maternal 25(OH)D levels regardless of pregnancy stage, so results should be cautiously evaluated. In their large prospective sub-cohort study comprising of 3658 Chinese pregnancies, Chen et al. [96] found a positive correlation between maternal serum 25(OH)D levels and neonatal birth weight. After adjustment for cofounders, the relative risk for low birthweight neonate and SGA was 12.31 (95% CI: 4.47, 33.89) and 6.47 (95% CI: 1.27, 3.13) among Vit-D-deficient mothers, and 3.15 (95% CI: 1.06, 9.39) and 2.01 (95% CI: 1.28, 3.16) among Vit-D-insufficient mothers, respectively. Another prospective study from the same geographical area included 3080 pregnancies and demonstrated that early pregnancy low levels of maternal Vit-D increase the risk of spontaneous abortion, as well as the risk of an SGA neonate [97]. However, the inclusion of subjects with gestational pathology might have introduced biases, as studies report increased bone resorption rates—in both mothers and fetuses—in pregnancies complicated with preeclampsia that may have influenced results [14,98,99]. In the tropical climate of Brazil, Pereira-Santos et al. [100] prospectively recruited 327 pregnancies earlier than 34 weeks of gestation. Measuring maternal 25(OH)D levels—without specifying the timepoint of sample collection—they reported a direct, significant association of maternal Vit-D levels with neonatal birth weight. In fact, each nmol increase in maternal Vit-D concentration resulted in an increase in birth weight of 3.06 g.

#### 3.2.5. Randomized Control Trials and Systematic Reviews

So far, study results regarding maternal Vit-D levels and intrauterine fetal growth seem to be inconsistent. Attempting to synthesize results from observational studies, researchers had to turn to metanalyses and systematic reviews of existing literature. A metanalysis of 16 studies examining the possible connection between maternal Vit-D deficiency and neonatal low birth weight was conducted recently by Fang et al. [101]. Researchers concluded that maternal Vit-D deficiency led to an increased risk of a low birthweight neonate (OR = 2.39, 95% CI: 1.25–4.57). However, neonates of deficient mothers had a lower birthweight of only 0.08 kgr (95% CI: −0.10 to −0.06) compared to controls, in agreement with a prior metanalysis [102]. Another metanalysis of 54 eligible studies showed that mothers with Vit-D levels lower, rather than higher, than 30 nmol/L, had newborns of lower birthweights and head circumferences, while no differences were found regarding infant birth length. When maternal Vit-D threshold for deficiency was raised to 50 nmol/L or 75 nmol/L, no significant differences were recorded. However, mothers with levels lower than 50 nmol/L had a raised risk for SGA and preterm birth. Furthermore, the offspring of Vit-D-deficient mothers had lower scores in the mental development test, while no differences were noted in language or motor development scores [103].

A recent Cochrane metanalysis reported that the clinical significance of the increased maternal serum 25(OH)D concentrations is still unclear [104]. However, women receiving vitamin D supplements during pregnancy less frequently had a baby with a birthweight below 2500 g than those receiving no intervention or placebo (RR 0.40; 95% CI 0.24 to 0.67, moderate quality). Furthermore, there was some indication that Vit-D supplementation increases infant length (mean difference 0.70, 95% CI −0.02 to 1.43) and head circumference at birth (mean difference 0.43, 95% CI 0.03 to 0.83) [104]. In a metanalysis of eligible studies, Vit-D insufficiency was associated with increased risk of SGA infants [105], while Vit-D supplementation during pregnancy was associated with increased circulating 25(OH)D levels, birth weight, and birth length [106]. Lastly, the recent systematic review by Pérez-López et al. [106] concluded that Vit-D supplementation during gestation had a positive impact on maternal Vit-D serum concentrations, as well as on neonatal birthweight and birth length, whereas no impact of 25(OH)D supplementation was observed on the incidence of SGA.

The obvious contrasting results reported by several studies regarding the possible correlations of maternal Vit-D levels with newborn anthropometric measures may not be only due to differences in study or sampling methods used, different ethnic groups with variable exposure to sunlight, and differences in maternal characteristics. The vast majority of the human studies reviewed herein—although indicative of possible correlations—are by nature observational, and thus with little potential to establish causal relations, so their results should be interpreted with caution. Given that Vit-D is either synthesized by human skin cells or obtained through diet, its levels may also reflect a crude measure of maternal nutritional status. For example, populations of low socioeconomic status residing in developing countries seem to have high Vit-D deficiency rates, as multiple studies report [37,44,86,88]. Furthermore, maternal hyperphagia or sub-optimal nutrition during gestation can also influence fetal growth and neonatal anthropometric indices, as well as fat deposition in offspring irrespectively of maternal Vit-D status [107].

On the other hand, interventional studies can also be found in the literature regarding maternal Vit-D supplementation, which seems to reduce the risk of a small-for-gestational-age neonate [104]. The underlying mechanisms are nevertheless still largely unknown, with current knowledge suggesting that there is no evidence to date to support the notion that maternal Vit-D supplementation during pregnancy has tangible effects on fetal growth parameters [104,108]. Moreover, there seems to be a well-established variation in birth weight across seasons, which could—at least partly—be explained by seasonal fluctuations in UV light exposure and Vit-D concentrations in maternal serum [108,109].

It is known that levels of 25(OH)D regulate bone homeostasis and mineral metabolism in humans. Its binding with the intracellular Vit-D receptor (VDR) acts as a gene transcription factor, ultimately promoting an increased bone mineral supply via intestinal calcium and phosphorus absorption and nephron calcium retention [110,111,112,113]. Furthermore, acting directly on the growth plate of long bones, it promotes bone maturation and calcification and activates both osteoblasts and osteoclasts, resulting in enhanced cellular bone formation and remodeling [112,113]. Due to vitamin’s D pivotal role in bone turnover and its multifaceted non-skeletal effects on immune and inflammatory responses [110], as well as on glucose metabolism and insulin sensitivity [114], we hypothesized that maternal Vit-D may affect fetal growth and development, and thus we included it in our systematic literature review.

Fang et al. [101], in their aforementioned systematic review and metanalysis, suggested several possible mechanisms that may underlie the presumable effects of maternal Vit-D status on fetal growth observed in some studies. Firstly—as already discussed—maternal Vit-D deficiency interferes with calcium absorption and affects bone metabolism, resulting possibly in reduced fetal bone mineralization in the fetal compartment. Secondly, there are indications in literature suggesting that Vit-D is involved in the regulation of the immune response in the fetal–maternal interface. Recently, it has been proposed that the anti-inflammatory properties of Vit-D may modulate inflammatory responses, leading to improved islet–cell functions and insulin release diminishing insulin resistance, while Vit-D deficiency may be a predisposing factor to impaired glycemic control and onset of Type 2 diabetes mellitus [67,115,116]. Sufficient Vit-D levels also seem to promote the production of antimicrobial molecules through toll-like receptor pathways, whereas its deficiency may lead to increased infection susceptibility and oxidative stress [117,118]. Others reported that cord blood Vit-D status in humans mediates innate immune response, while its deficiency can alter monocyte responses in vitro, resulting in defects in antimicrobial barrier protection against pathogens [119].

Lastly, Vit-D and its metabolites may be implicated in the regulation of metabolism of a variety of other molecules, such as glucose, insulin growth factor 1, and fatty acids, possibly affecting the supply of nutrients to the developing fetus via the placenta [120]. Moreover, a previous study showed that increased levels of Vit-D enhance the invasion of human extravillous trophoblast, with authors postulating that Vit-D sufficiency may have a preventive effect on the onset of endothelial damage and preeclampsia in human gestation [121]. Indeed, mechanistic evidence for Vit-D effects on angiogenesis have been found in conjunction with an upregulation of angiogenic factor vascular endothelial growth factor (VEGF) [122]. These altered angiogenic pathways, in combination with the correlation of human placental gene expression of amino acid transporters with maternal Vit-D status and Vit-D binding protein reported by Cleal et al. [123], imply that Vit-D indeed mediates fetal nutrient supply via the placenta [124]. Adding more complexity to these biological mechanisms, Ashley et al. demonstrated experimental data of an active placental uptake of maternal 25(OH)D_3_ placental metabolism into active metabolites and their subsequent active release into both maternal and fetal compartments, suggesting that fetal Vit-D supply is not only dependent on its availability to the placenta, but also on placental function itself [125,126].

#### 3.2.6. Maternal Sclerostin Levels during Pregnancy and Anthropometrics at Birth

Data in the international literature concerning maternal bone metabolism molecules, apart from Vit-D, in relation to offspring birth anthropometrics, are sparse (Supplemental Table S3). Concerning maternal sclerostin concentrations, Briana et al. studied prospectively 80 full-term pregnancies in Greece during parturition. After grouping maternal-neonatal pairs in three categories depending on neonatal birthweight (30 intrauterine growth restricted, 30 LGA, and 20 appropriate-for-gestational-age neonates), they found no differences in maternal serum sclerostin levels among the three groups [127]. However, maternal serum sclerostin levels were higher in older mothers and in cases complicated with GDM in the LGA group, with authors suggesting a possible role of elevated sclerostin levels in poor bone quality and increased bone fragility in women suffering from diabetes. The above results oppose those of the beforementioned study by Mastorakos et al. [49], who demonstrated a positive correlation between maternal sclerostin levels and neonatal birthweight. Nevertheless, data should be carefully interpreted, as Briana et al.‘s study included mothers with various pathological conditions in the IUGR and LGA group, that may have influenced the outcome.

In another prospective study, Godang et al. concluded that maternal sclerostin levels do not have a significant role in neonatal bone mass [128]. Studying 202 pregnancies between 30–32 weeks of gestation, they found no correlation between maternal serum sclerostin levels and bone mineral content at birth in offspring, as measured by dual-energy X-ray absorptiometry, whereas umbilical cord plasma sclerostin levels were significantly associated with neonatal bone mineral content, even after adjustment for covariates [128]. Other maternal bone metabolism biomarkers studied (a-klotho, fibroblast growth factor 23, and 25(OH)D), showed no significant correlations either.

#### 3.2.7. Maternal Osteocalcin Levels during Pregnancy and Anthropometrics at Birth

Regarding maternal osteocalcin levels during pregnancy and their possible effects on neonatal anthropometry, we were able to detect only one relevant study in our review. In their nested case-control study, Wei et al. [129] investigated maternal osteocalcin levels throughout all three trimesters of pregnancy, in relation to low birthweight infants in China. They demonstrated that mothers with elevated osteocalcin serum levels during the first and second trimesters had a 2.3- and 1.59-times higher risk of a low-birthweight neonate respectively, compared to subjects with low maternal osteocalcin levels, even after adjustment for confounders. However, no associations were noted regarding either maternal osteocalcin levels during third trimester, or maternal 25(OH)D concentrations during gestation and low birthweight. Lastly, the authors proposed that maternal osteocalcin may act as a compensatory mechanism to enhance fetal glucose uptake in low-birthweight pregnancies through insulin secretion and sensitivity adjustment, ultimately promoting brain development.

#### 3.2.8. Maternal Osteoprotegerin Levels during Pregnancy and Anthropometrics at Birth

The work of Briana et al. on 40 full-term pregnancies found no significant differences in maternal serum osteoprotegerin and sRANKL levels during the first stage of labor, between mothers of intrauterine growth-restricted, and appropriate-for-gestational-age neonates [21]. On the other hand, Essley et al. [22], studying prospectively 155 adolescent pregnancies during mid-gestation, reported a significant correlation between maternal and neonatal cord blood osteoprotegerin levels. In turn, neonatal cord blood osteoprotegerin concentration at birth was significantly and inversely correlated with both neonatal birth weight z-score and ponderal index. Nevertheless, no direct correlations between maternal osteoprotegerin levels and neonatal birthweight or ponderal index were reported.

Although there is still limited data on humans, experimental and clinical studies in animal models suggest the emerging endocrine role of sclerostin in regulating fat metabolism [50]; sRANKL on the other hand, has been shown to participate in an interplay between fetal and maternal compartments in mice, thus promoting maternal tolerance of the conceptus [130]. Furthermore, the sRANKL inhibition in osteoporotic patients leads to insulin sensitivity improvement [52], implying its role in glucose metabolism—the main metabolic fuel for fetal development—and indirectly in excess fetal adipose tissue deposition. All the above indications existing in the literature have yet to be validated in human gestation in order to establish causal effects of maternal bone turnover molecules on fetal growth, however we believe that the current manuscript will trigger further investigation into this matter.

### 3.3. Maternal Bone Turnover Molecules during Pregnancy and Intrauterine Fetal Glucose Metabolism and Insulin Resistance

Current evidence suggests that bone can act as an endocrine organ, as various bone turnover molecules are implicated in energy equilibrium processes, with the exact biological mechanisms remaining largely unknown to this date. These mechanisms are likely to be of significance in fetal development during human gestation, given the emerging role of these molecules in maternal glucose homeostasis—the main energy substrate for intrauterine growth development—and fetal adipose tissue accumulation. Intrauterine and neonatal life is a rather sensitive period of development and some of the presumable effects of the maternal bone molecules in question may lead to in utero alterations in fetal measures of glucose homeostasis. Therefore, in this section, we sought to review the literature regarding possible, measurable alterations in levels of fetal metabolic indices in amniotic fluid or cord blood, which could be correlated with maternal bone turnover status during pregnancy. For example, it has been postulated that β-cell destruction in cases of Type 1 diabetes mellitus may occur during intrauterine life [131], while Vit-D deficiency is associated with a diminished β-cell action in preliminary studies [132]. Since fetal 25(OH)D levels depend mainly on maternal compartment, it would be of value to investigate for possible associations of maternal Vit-D status with measures of intrauterine β-cell function.

However, the literature contains only a few studies of amniotic fluid or cord blood metabolic indices in relation to maternal bone marker molecules, summarized in Supplemental Table S4. In a sub-analysis of HAPO study at the Belfast center including 1150 pregnancies, Casey et al. [66] reported no significant associations between maternal 25(OH)D levels at 28 weeks and cord blood glucose levels, HOMA-IR and HOMA-β at delivery, in both the unadjusted and adjusted analysis. In another study, researchers assessed C-peptide concentrations in umbilical cord plasma in 202 neonates at birth, as C-peptide levels can be used as an index of fetal beta-cell function. They reported no significant associations between maternal 25(OH)D concentrations and C-peptide levels [76]. Lack of any associations persisted after adjustment for maternal BMI. Lastly, our search did not reveal any relevant studies to date concerning maternal levels of sclerostin, RANKL, osteoprotegerin, or osteocalcin and fetal intrauterine metabolic indices.

### 3.4. Maternal Bone Turnover Molecules during Pregnancy and Possible Metabolic, Endocrine, and Immunological Consequences in Offspring

Life in utero is considered a crucial period of human development, in terms of neonatal developmental programming of future health and disease. However, only a few studies to date concentrated on the investigation of possible effects of maternal bone turnover molecules on later health of offspring (Supplemental Table S5). Studies in rodents for example, suggest that maternal osteocalcin crosses the placental barrier and inhibits fetal neuronal apoptosis, influencing cognition in adult offspring [133]. Whether similar effects of osteocalcin or other maternal molecules involved in bone metabolism are in place during human pregnancy remains to be investigated.

Maternal Vit-D on the other hand, has been more extensively studied. A growing body of literature suggest that low maternal serum 25(OH)D concentrations during human gestation are associated with accelerated childhood growth patterns. In their prospective study, Neelon et al. [134] demonstrated that reduced 25(OH)D levels in maternal serum during early to mid-gestation are associated with higher 1-year weight-for-length, as well as 3-year BMI z-scores in offspring. Similarly, in the large multiethnic study of Leffelaar et al. [56], children of early pregnancy Vit-D deficient mothers showed accelerated growth in both weight and length at 6 and 9 months of life, regardless of maternal ethnicity. Moreover, infants in the deficient category were significantly smaller in terms of length at month 1, but significantly larger at 12 months of age. These results partly validate previous research; Eckhardt et al. [71], retrospectively studying maternal Vit-D levels in 2473 multiethnic pregnancies earlier than 26 gestational weeks, reported that infants of mothers with 25(OH)D levels higher than 30 nmol/L had 0.13- and 0.20-units higher z-scores for length and head circumference respectively across the first year of life. However, by 12 months of age, these differences were resolved, suggesting steeper growth trajectories in infants with maternal 25(OH)D levels lower than 30 nmol/L. On the other hand, another recent study from China did not find any associations between maternal Vit-D concentrations and offspring BMI-z trajectories during 0 to 3 years of life [135].

A few studies have also examined the possible relation of maternal Vit-D status with offspring body composition. In a large Spanish prospective study, Morales et al. [41] demonstrated that maternal Vit-D deficit during early second trimester is associated with increased risk of an overweight offspring at age 1 year. Nevertheless, this association faded by 4 years of age. The findings of the Southampton Women’s Survey [136] pointed in the same direction: lower maternal Vit-D status at 34 weeks was associated with lower neonatal fat mass at birth, but with greater fat mass at 4 and 6 years of age. Recently, Hyde et al. reported that—in smokers only—higher maternal 25(OH)D before 16 weeks is associated with lower offspring fat and higher mean lean mass percentage at the 11-year follow up. A similar trend was evident also in non-smokers, while no associations were found regarding maternal 25(OH)D at 28–32 weeks and offspring body composition [16]. Another study from an Indian cohort of 568 mother–offspring pairs further supports a link between maternal Vit-D status during 28 to 32 weeks of gestation and offspring fat mass. This study showed that boys born to mothers with 25(OH)D deficiency had a higher body fat percentage and lower fat-free mass compared to girls at the age of 5 years, but not at 9.5 years [137]. Children of Vit-D deficient mothers had also significantly smaller arm–muscle area (as a measure of muscle mass) compared to children born to mothers with normal Vit-D levels at both 5- and 9.5-years follow-up, independently of sex. Although the study reported no differences in cardiovascular risk markers between children of Vit-D deficient and sufficient mothers at the age of 5, lower maternal 25(OH)D status was associated with higher insulin resistance at the age of 9.5 years, even after data correction for all maternal covariates. Furthermore, higher HDL-cholesterol levels were observed among 9.5-year-old boys in the maternal Vit-D deficiency group [137]. Besides, a study of 160 participants in UK demonstrated that mothers with lower serum 25(OH)D levels during late pregnancy had children with a deficit in bone-mineral accrual which persisted to the age of 9 years, possibly raising the risk for osteoporotic fractures in later life. The deficit was manifested as reduced whole body and lumbar spine bone-mineral content, without effects on childhood height or lean mass [138].

On the other hand, Gale et al. in their prospective study in UK, reported no statistically significant associations between maternal 25(OH)D concentrations at late pregnancy and offspring anthropometrics or cardiovascular risk indices at 9 months and 9 years of age, although weight, fat mass and lean mass tended to be lower in children of mothers in the lowest 25(OH)D quartile in 9-year-olds. Nevertheless, 9-year-old children of mothers belonging to the higher-serum Vit-D quartile had a significantly larger head circumference compared to those of mothers with lower 25(OH)D concentrations [139]. Interestingly enough, children of mothers with 25(OH)D concentrations over 75 nmol/L demonstrated an increased risk of eczema at 9 months (OR 3.26, 95% CI 1.15–9.29, *p* = 0.025) and asthma at age 9 years (OR 5.40, 95% CI, 1.09–26.65, *p* = 0.038) compared to their counterparts born to mothers with 25(OH)D concentrations lower than 30 nmol/L [139]. Similarly, a more recent large multiethnic study of 910 participants in Singapore reported no statistically significant associations between maternal Vit-D status at 26–28 weeks and any of the following outcomes: SGA (OR 1.00; 95% CI 0.56, 1.79), weight-for-age z-scores, length-for-age z-scores, circumferences of head, abdomen and mid-arm, BMI, and skinfold thickness (triceps, biceps, and subscapular) at birth or during the first two years of life. However, researchers underlined the very low prevalence of severe maternal Vit-D deficiency in their sample (1.6%) [140]. Another prospective cohort study from Denmark investigated the possible associations between maternal Vit-D status at 30 gestational weeks and cardiometabolic health in offspring at 20 years of age [141]. Authors reported no correlations between maternal Vit-D status and BMI or waist circumference in the 20-year-old offspring, independent of sex. No association was also demonstrated neither with glucose metabolism parameters measured, such as fasting glucose, insulin, HOMA-IR, and HbA1c levels, nor with lipid metabolism indices (total and LDL cholesterol, triglycerides, and apolipoprotein B) or blood pressure. Nevertheless, a weak inverse association between maternal 25(OH)D and HDL cholesterol was found in females.

There is also increasing evidence in literature regarding the association of maternal bone turnover molecules during human gestation with immunological status of offspring. A recent randomized control trial by Hornsby et al. [142] compared the effects on neonatal immunity of maternal 25(OH)D_3_ daily supplementation with 4400 IU or 400 IU during second and third trimesters, analyzing newborn cord blood samples. They found that maternal Vit-D3 supplementation with 4400 IU daily resulted in a significantly enhanced proinflammatory cytokine production in response to innate and mitogenic stimuli, greater IL-17A production in response to T-cell stimulation, and greater dexamethasone-induced IL-10 production. The authors concluded that, given the evidence for strong neonatal immune responses in early life being associated with decreased odds of asthma development, high levels of maternal Vit-D supplementation can contribute to protection from asthma and other asthma-related infectious conditions in early life [142].

Given the evident involvement of Vit-D in modulation of immune responses, Zosky et al. [143], in their retrospective study, investigated the possible associations between maternal Vit-D status during 16 to 20 weeks of gestation with the onset of atopy, eczema, and wheeze in offspring at the age of 6 and 14 years. They reported no associations between maternal Vit-D status and atopic status in offspring. Contrarily, maternal 25(OH)D deficiency (<50 nmol/L) was associated with wheeze and asthma (evident only in boys) at the age of 6 years. These associations attenuated by 14 years of age. However, previous results by Gale et al. [139] suggested a trend towards an increased risk of atopic disorders in mothers with 25(OH)D levels exceeding 75 nmol/L, while Pike et al. [144] concluded that there is no evidence towards a greater risk of childhood asthma, wheeze, or atopy in children of mothers exposed to increased 25(OH)D concentrations during late pregnancy. Morales et al. [145], on the other hand, concluded that higher maternal 25(OH)D levels were independently associated with lower risk of respiratory tract infections in their children during the first year of life, but no connections were noted with childhood asthma or wheezing while others found no correlations between maternal Vit-D at parturition and odds of respiratory tract infections in the first year of life [91]. These conflicting results may be attributed to the different timepoints in pregnancy that Vit-D concentrations were assessed, suggesting a variable impact of maternal Vit-D status on postnatal lung functionality, depending on the fetal developmental stage. Another study by Erkkola et al. [146] investigated maternal Vit-D dietary intake during pregnancy in relation to asthma and allergic rhinitis onset in 5-year-old offspring. They reported that—after correcting data for covariates—maternal Vit-D dietary intake was negatively associated with the risk of asthma and allergic rhinitis onset. Nevertheless, data derived solely from questionnaires in this study and maternal Vit-D levels were not measured.

Type 1 diabetes mellitus is one of the most common autoimmune diseases during childhood. Given the effects of Vit-D in biological processes of inflammation modulation, researchers investigated whether maternal Vit-D status during pregnancy is associated with the onset of type 1 diabetes mellitus in the offspring. Preliminary data from a nested case-control study in Norway including 109 mothers of children who later developed type 1 diabetes, suggested that there is a trend toward a two-fold higher risk of onset of the disease in the offspring of mothers with their late pregnancy 25(OH)D levels belonging to the lowest quartile, compared to children of mothers with levels above the upper quartile [147]. The same researchers had previously published that maternal use of Vit-D rich cod liver oil during pregnancy was less frequent in cases of childhood-onset type 1 diabetes than in control subjects [148]. Other studies also reported a reduced diabetes-related autoimmunity at age 1, but not at later ages, among children of mothers using Vit-D supplements during pregnancy [149], and even a protective role of Vit-D intake against the appearance of autoimmunity in offspring [150]. However, a similar Finnish study did not find such associations with either autoantibodies or diabetes mellitus in the offspring of mothers using Vit-D supplementation during pregnancy [151]. In accordance were the results reported by Miettinen et al. [152]; comparing retrospectively maternal serum levels of 25(OH)D during early gestation, between 343 mothers of children who later developed type 1 diabetes and 343 mothers of healthy children in Finland, they found no significant differences in maternal 25(OH)D levels among children who later developed type 1 diabetes and their same-age, healthy counterparts. However, the mean age at diabetes diagnosis was 3 years, many years before the average age of the disease diagnosis [153]. Lastly, a recent systematic review and metanalysis of two relevant studies concluded that maternal Vit-D deficiency during pregnancy was not associated with the risk of diabetes type 1 development in the offspring (OR 1.25, 95% CI 0.78, 2.02) [102].

## 4. Conclusions

Our study sought to systematically review current knowledge on to what extent maternal bone turnover molecules during pregnancy influence intrauterine fetal development and metabolism, as well as offspring future metabolic and endocrine wellbeing. Although Vit-D remains the most extensively studied bone turnover biomarker during human pregnancy, the emerging role of other maternal molecules involved in bone metabolism (such as osteocalcin, sclerostin, RANKL, and osteoprotegerin) in endocrine and metabolic pathways in offspring, gains more and more attention in the recent years, but—although promising—research is still in early stage. Via this systematic review, we sought to attract the attention of the scientific community on the emerging endocrine multifaceted roles of bone metabolism molecules during human gestation and their possible influence on the developing fetus and to provide a basis which will prompt for further research.

Concerning maternal Vit-D, we observed a large heterogeneity of studies. These discordant results can be explained, at least partly, by the large variability in study methods and 25(OH)D cutoffs, time of sampling during pregnancy, seasonal variations of Vit-D levels and the large heterogeneity of population under study (due to different culture, economic levels, supplement use, dietary patterns, maternal BMI, gene polymorphisms of Vit-D receptor, latitude, gestational complications, and sample sizes) [9,14,35,101,103,135]. As a result, studies on the effects of maternal Vit-D on intrauterine fetal growth and development are still mainly inconclusive, however there seems to be a trend of a negative Vit-D deficiency impact on fetal growth, although not evident in all studies. It must be noted, however, that in utero fetal growth is assessed by measuring ultrasound indices with great inter- and intra-observer variability. Furthermore, it is not clear which measurements are influenced by maternal Vit-D concentrations or best reflect intrauterine growth, due to variations in fetal body composition.

Early days in research show maternal sclerostin and sRANKL to correlate positively with fetal abdominal circumference and subcutaneous fat deposition in second trimester, contributing to greater birthweights. Regarding anthropometric measures at birth, studies, as well as their systematic reviews and metanalyses, seem to conclude that severe maternal Vit-D deficiency is generally related with lower birthweights and a higher risk of an SGA neonate—especially in cases complicated with maternal overweight or obesity [101,103]. Other bone turnover biomarkers also seem to impact fetal growth; maternal serum sclerostin was positively associated with neonatal birthweight, while in another study, elevated maternal osteocalcin levels were associated with an increased risk of a low-birthweight newborn. Maternal osteoprotegerin correlated positively with cord blood osteoprotegerin but was not directly associated with anthropometric measures in offspring, while cord blood osteoprotegerin significantly and inversely correlated with neonatal birthweight z-score and ponderal index. Herein, we underline the lack of studies in the literature on the effect of maternal bone turnover molecules on intrauterine fetal metabolism, despite their emerging role in endocrine and metabolic processes. Nevertheless, the only two available studies reviewed here did not find any relations.

Currently there also seems to be a gap in knowledge regarding the effects of maternal bone molecules during pregnancy on future metabolic and endocrine health of offspring. However, maternal 25(OH)D deficiency during gestation has already been linked with accelerated, catch-up growth patterns during early childhood, which in turn have been linked with childhood adipose tissue accumulation and a higher risk of subsequent overweight or obesity [154,155]. Indeed, some of the studies reviewed herein suggest that neonates of Vit-D deficient mothers seem to have a higher risk of being overweight and having a higher percentage of fat mass during the first years of life. Nonetheless, the existing literature suggests that there are no associations between low maternal Vit-D levels during gestation and future cardiovascular, glucose or lipid metabolism sequalae in offspring to date. On the other hand, some studies demonstrated a possible trend, linking maternal Vit-D deficiency with a greater risk of Type 1 diabetes mellitus development in the offspring. Such results should be cautiously evaluated; nevertheless, they warrant further research.

From the current literature, it becomes apparent that maternal bone turnover molecules are likely to be implicated in intrauterine fetal growth, neonatal birthweight, and at least to some extent, in childhood body composition and metabolic wellbeing. Nonetheless, the exact biological mechanisms involved remain largely elusive. A growing body of literature supports the existence of an intriguing interplay between bone, glucose metabolism, and adipose tissue. However, current data seem insufficient to implement new clinical guidelines. Concerning maternal Vit-D, researchers and clinicians should focus on achieving an international consensus on the definition of Vit-D status, which probably should be personalized according to ethnicity, season of childbearing, and stage of gestation. More randomized control trials in large samples are needed to determine the maternal group which will most benefit by Vit-D supplementation in the most effective way—regarding dosing and timing of gestation—in order to diminish the risks of a low birthweight or SGA. Lastly, as previously described, given the sex-specific rates of intrauterine growth, which seem to render male fetuses more vulnerable to maternal insufficiencies than females during gestation [107,156], new studies should also focus on sex-dependent fetal growth effects of maternal bone metabolism, as suggested by some of the studies [72,73,94,137,141].

## Supplementary Materials

The following supporting information can be downloaded at: https://www.mdpi.com/article/10.3390/ijms23158328/s1.
