# Supplementary material for: The Gestational Effects of Maternal Bone Marker Molecules on Fetal Growth, Metabolism and Long-Term Metabolic Health: A Systematic Review
Source: Int J Mol Sci. 2022 Jul 28;23(15):8328. doi: 10.3390/ijms23158328 (PMC9368754; doi:10.3390/ijms23158328)
Supplement: Supplementary file 1 [file ijms-23-08328-s001.zip › Table S1_Ultrasound Indices.pdf]

Author	Type of study	N	Ethnicity	Marker studied	Trimester of serum measurement	Marker Cutoffs	Adjusted for covariates	Results	Others
Zhang et al.	retrospective cohort study	15651	China	25(OH)D	1st trimester	Vit D sufficiency > 50 nmol/L<br>Vit D insufficiency/deficiency < 50 nmol/L	-	In Vit D insufficiency/deficiency group, CRL was decreased (P = 0.005), and the risk of early FGR was increased by 13% (95% CI 1.04–1.24, P = 0.004) compared to the Vit D sufficient group.<br><br>There was a significant combined effect of maternal Vit D concentrations and overweight/obesity on CRL (P for interaction = 0.02) and the risk of early FGR (P for interaction = 0.07).	
Aydeniz et al.	retrospective, cross sectional	154	Turkey	25(OH)D3	1st trimester (12-14 w)	Group 1 <10 ng/ml<br>Group 2 >10–16 ng/ml<br>Group 3 >16–20 ng/ml<br>Group 4 >20–30 ng/ml<br>Group 5 >30 ng/ml.	-	No relationship between maternal vitamin D levels and neonatal birth weight and length.	
Judistiani et al.	prospective	203	Indonesia	Vitamin D	1st	deficient < 20 ng/mL)<br>insufficient 20–29.99 ng/mL<br>normal ≥30 ng/mL).	maternal age, pre-pregnancy body mass index, parity, serum ferritin level, and hemoglobin level	Maternal serum vitamin D showed significant associations with biparietal diameter (β = 0.141, p = 0.042) and abdominal circumference (β = 0.819, p = 0.001) after adjustment with	
Lee et al.	prospective	245	Korean	25(OH)D	12-14<br>20-22<br>32-34 w<br>(VitD and ultrasounds)	deficiency <20 ng/mL	-	Negative correlation between serum 25(OH)D level at 12-14 weeks and growth velocity of fetal biparietal diameter between 20-22 and 32-34 weeks.<br>Positive correlation between the difference of serum 25(OH)D levels between 12-14 and 20-22 weeks and growth velocity of fetal biparietal diameter between 20-22 and 32-34 weeks<br><br>The changes of 25(OH)D levels between 12-14 and 20-22 weeks are important for growth of fetal biparietal diameter	
Morales et al.	prospective	n=2221<br>Femur length	Spain	25(OH)D3	13-15 w	deficit <20 ng/mL	-	Maternal deficit of 25(OH)D3 was associated with increased risk of fetal	

		n=2166<br>Biparietal diameter<br><br>n=2210<br>Estimated fetal growth<br><br>n=2219<br>Abdominal circumference						overweight defined as AC ⩾ 90th percentile (odds ratio (OR) = 1.50, 95% confidence interval (CI): 1.01–2.21; P = 0.041) or either as EFW ⩾ 90th percentile (OR = 1.47, 95% CI: 1.00–2.16; P = 0.046).<br><br>Deficit of 25(OH)D3 was associated with an increased risk of overweight in offspring at age 1 year (OR = 1.42, 95% CI: 1.02–1.97; P = 0.039).<br>The association was attenuated at age 4 years (OR = 1.19, 95% CI: 0.83–1.72; P = 0.341).
Walsh et al.	prospective	60	Ireland	25(OH)D	Early gestation and at 28w<br><br>U/S at 20 and 34 weeks	sufficient > 50 nmol/L<br>at high risk of deficiency <30 nmol/L	-	Winter cohort: correlation between early pregnancy 25(OH)D and FL at 20w (r=0.34, P=.07) & between 28-week 25(OH)D and FL at 34w (P=.02).<br>Infant length was shorter in mothers with early pregnancy 25(OH)D levels below median (52.1 vs. 53.6 cm, P=.04)<br><br>There are marked seasonal variation of 25(OH)D levels
Fernández-Alonso et al.	Cross sectional	498 (CRL)<br>487 (NT)	Spain	25(OH)D	1st trimester	adequate >30 ng/mL<br>insufficient 20–29.99 ng/mL<br>deficient <20 ng/mL)	-	No correlation with CRL and NT measurements
Marçal et al.	cross-sectional study	87	Brazil	25(OH)D	26-36w	Deficiency <20ng/mL (50nmol/L)<br>insufficiency 20 - 30ng/mL (50–75nmol/L)<br>sufficiency 30–100ng/mL (75–250nmol/L)	-	No differences in maternal vit D among pregnant women with AGA, SGA, or FGR fetuses (r= -0.06, p=0.551)
Liu et al.	Prospective cohort	10913	China	25(OH)D	24-28 w	Deficiency < 20 ng/mL	-	Maternal 25(OH)D deficiency and GDM were independently associated with an increased risk of excessive fetal growth. The combination of the two was associated with an increased risk of excessive fetal growth assessed by EFW Z-score (OR: 1.36, CI: 1.15-

								1.62) and AC Z-score (OR: 1.32, CI: 1.11-1.56)<br><br>Maternal 25(OH)D deficiency and GDM may jointly increase the risk of excessive fetal growth.
Sarma et al.	prospective cohort	250	India	25(OH)D	3rd trim.	Deficiency <20 ng/ml<br>insufficiency 21-29 ng/ml	-	Fetal femur length and birth length were significantly shorter in mothers with low Vitamin D (r = 0.60, P < 0.01 and r = 0.611, P < 0.01 respectively)<br><br>No significant associations were noted between birth weight and head circumference and maternal Vitamin D levels
Young et al.	Prospective longitudinal	171 adolescents	USA	25(OH)D & 1,25(OH)2D	Mid-gestation (26 w)	insufficiency <50 nmol/L	Maternal smoking, height, race, weight gain, and gestational age	Maternal 25(OH)D sufficiency was positively associated with fetal femur and humerus z-scores (P<0.01) even after adjustment for co-factors<br>Optimal calcium intake and adequate maternal vitamin D status are both needed to maximize fetal bone growth
Ioannou et al.	prospective	357	UK	25(OH)D	34w	insufficiency <75 nmol/L<br>sufficiency >75 nmol/L	maternal age, height, and smoking	25(OH)D levels correlated with femoral volume (P=0.006; r=0.147) and proximal metaphyseal diameter (PMD) (P=0.001; r=0.176)<br><br>On multiple regression only the effect of 25(OH)D on PMD remained significant
Mahon et al.	prospective	424	UK	25(OH)D	34 w	sufficient/borderline >50 nmol/L<br>insufficient 25-50 nmol/L<br>deficient ≤25 nmol/L	-	Lower 25(OH)D was not related with FL but was associated with greater femoral metaphyseal cross-sectional area and a higher femoral splaying index at 19 weeks (r=−0.16, (95%CI:−0.25 to −0.06) and r=−0.17, (95%CI: −0.26 to −0.07), respectively and at 34 weeks gestation (r=−0.10, 95%CI:−0.20 to 0.00) and r=−0.11, (95%CI: −0.21 to −0.01), respectively
Miliku et al.	population-based, prospective cohort	7098	Netherlands	25(OH)D	2nd trim.	quartile 1: 1.5–24.1 nmol/L<br>quartile 2: 24.2–46.6 nmol/L	-	Mothers who had 25(OH)D concentrations in the lowest quartile had an increased risk of SGA (OR: 2.07; 95% CI: 1.33, 3.22).

						quartile 3: 46.7–73.7 nmol/L<br>quartile 4: 73.8–193.2 nmol/L		Neonates born to mothers with 25(OH)D levels belonging to the two lowest quartiles had restricted head circumference from 2nd trim, onward [differences: 1st quartile -0.20 SDS (95% CI: 20.29, 20.12 SDS) and 2nd quartile -0.09 SDS (95% CI: 20.17, 20.01 SDS)]<br><br>Low maternal 25(OH)D concentrations are also associated with an increased risk of preterm birth and small size for gestational age at birth.
Galthen-Sørensen et al.	Systematic review	5 observational studies	-	25(OH)D	-	-	-	low maternal 25(OH)D levels may affect fetal bone growth under certain circumstances, especially in cases of simultaneous low calcium intake
Mastorakos et al.	prospective	100	Greece	Sclerostin, sRANKL, osteocalcin, 25(OH)D3	All trimesters	-	gestational age, maternal BMI, maternal insulin concentrations, fetal sex	2nd trim.: maternal sclerostin positively associated with abdominal circumference (r=0.361, P=0.03) and birthweight (r=0.632, P<0.001), while maternal sRANKL with fetal abdominal subcutaneous fat thickness (r=0.357, P=0.034), sagittal abdominal diameter (r=0.502, P=0.004) and abdominal circumference (r=0.416, P=0.015)<br>Maternal 25(OH)D3 correlated positively with fetal abdominal subcutaneous fat thickness (r=0.287, P=0.032) and neonatal abdominal circumference (r=0.344, P=0.407)<br>3rd trim.: maternal sclerostin correlated positively with fetal abdominal diameter (r=0.763, P=0.019), while sRANKL with fetal abdominal circumference (r=0.328, P=0.044) and sagittal abdominal diameter (r=0.402, P=0.038)<br><br>Maternal second trimester serum sclerostin levels were the best positive predictors of birth weight P=0.003, beta coeff. = 0.568, adj. R^2=0.264

Table S1: Studies on the effect of maternal bone turnover biomarkers on intrauterine fetal growth. CRL: crown-rump leght, FGR: fetal growth restriction, SGA: small for gestational age, EFW: estimated fetal weight, NT: nuchal translucency, AGA: appropriate for gestational age, LGA: large for gestational age, GDM: gestational diabetes mellitus
