# Supplementary material for: The Gestational Effects of Maternal Bone Marker Molecules on Fetal Growth, Metabolism and Long-Term Metabolic Health: A Systematic Review
Source: Int J Mol Sci. 2022 Jul 28;23(15):8328. doi: 10.3390/ijms23158328 (PMC9368754; doi:10.3390/ijms23158328)
Supplement: Supplementary file 1 [file ijms-23-08328-s001.zip › Table S2_VitD_Birth Anthropometrics.pdf]

| Author | Type of study | N | Ethnicity | Trimester | Adjusted for covariates | Maternal Marker Studied | Cutoff used | Outcome | Other outcomes/Comments |
|---|---|---|---|---|---|---|---|---|---|
| **Early gestation** | | | | | | | | | |
| ***Wang H.*** | prospective | 747 | China | 1st prenatal visit | maternal age, maternal race, pre-pregnancy BMI, height, smoking, planned pregnancy, marital status, education, family's socio-professional category, delivery, season, Vit D supplementation, physical activity, Vit D lifestyle score, and infant sex | 25(OH)D | Deficiency ≤20 ng/ml) insufficiency 20–29 ng/ml sufficiency ≥30 ng/ml | Reported a nonlinear relation between 25(OH)D, birth weight and head circumference (P < 0.01). Birth weight and head circumference increased by 69 (95% CI: 38 –122) g and 0.31 (0.22–0.40) cm, respectively, per 1 ng/ml increase in maternal 25(OH)D, up to 20 ng/ml and leveled off thereafter | For each 1 unit decrease of maternal 25(OH)D, the unadjusted and adjusted risk of SGA increased by 19% (odds ratio 1.19 [95% CI 1.13–1.25], P < 0.001) and 9% (1.08 [1.03–1.16], P = 0.009), respectively. |
| ***Fernando et al.*** | retrospective | 304 | Australia | Early pregnancy <20w | maternal covariates including age, body mass index (BMI) and ethnicity | Total, free and bioavailable 25(OH)D | - | Higher maternal total and free 25(OH)D concentrations were associated with higher neonatal birthweight (β = 5.05, p = 0.002 and β = 18.06, p = 0.02, respectively | |
| ***Hyde et al.*** | prospective | 402 | Australia | At booking <16 and 28-32 | Maternal smoking status | 25(OH)D | - | In smokers, but not non-smokers, serum 25(OH)D status at recruitment was inversely associated with newborn's fat mass percentage but positively associated with lean mass (both p < 0.05) | No association at 28–32 weeks of gestation |
| ***Leffelaar et al.*** | prospective | 3730 | The Netherlands (multiethnic) | 12-14w | fetal sex, maternal height, parity, maternal age, smoking, maternal pre-pregnancy | 25(OH)D | Deficient ≤29·9 nmol/l, insufficient 30–49·9 nmol/l | compared with women with adequate Vit D levels, women with deficient Vit D levels | Neonates born to mothers with a deficient Vit D status showed accelerated growth in weight and length during the first year of life |

| | | | | | | | | | |
|---|---|---|---|---|---|---|---|---|---|
| | | | | | BMI and educational level | | and adequate’≥50 nmol/l | had infants with lower birth weights (2114·4 g, 95 % CI 2 151·2, 2 77·6) and a higher risk of SGA (OR 2·4, 95 % CI 1·9, 3·2 | |
| ***van den Berg et al.*** | population-based birth cohort study | 2,274 | Netherlands | Early pregnancy | Maternal overweight, season of blood sampling, and maternal smoking | 25(OH)D | - | women in the lowest 25(OH)D quartile had a higher risk of an SGA offspring (OR 1.57, 95% CI 1.03–2.39)<br><br>25(OH)D appears to be a modifiable contributor to the association between low maternal education and SGA offspring, particularly in overweight women and women who conceived in the winter period | |
| ***Gernand et al.*** | retrospective | 2146 | USA multicentric | At booking and every 8 weeks after and up to 26 | season of blood sampling, gestational age, pre-pregnancy body mass index, height, parity, smoking, marital status, socioeconomic status, maternal age, study site (accounts for latitude), and infant sex | 25(OH)D | - | mothers with 25(OH)D of 37.5 nmol/liter or greater gave birth to newborns with 46 g [95% confidence interval (CI), 9–82 g] higher birth weights and 0.13 cm (0.01–0.25 cm) larger head circumferences compared with mothers with less than 37.5 nmol/liter<br><br>No association was observed between 25(OH)D and ponderal index, placental weight, or the placental to fetal weight ratio<br><br>Maternal 25(OH)D of 37.5 nmol/liter or greater vs. less than 37.5 nmol/liter in the first trimester was associated with half the risk of small for gestational age (adjusted odds ratio 0.5; 95% CI 0.3– 0.9), but no second-trimester association was observed | |
| ***Rodriguez et al.*** | Prospective cohort | 2382 | Spain | 11-15 w | child’s sex, gestational age, month at blood sampling, parity, maternal age, ethnic background, maternal social class and education level, maternal pre-pregnancy body mass index, | 25(OH)D3 | Insufficiency: 20–29.99 ng/ml] deficiency: <20 ng/ml], sufficiency: ≥30 ng/ml] | Offspring of mothers with higher circulating 25(OH)D3 concentration tended to have smaller HC [coefficient (SE) per doubling concentration of 25(OH)D3, 0.10 (0.05), P = 0.038]. | No evidence reported of an association between Vit D status in pregnancy and GDM, preterm delivery, FGR, SGA and anthropometric birth outcomes |

| | | | | | | | | | |
|---|---|---|---|---|---|---|---|---|---|
| | | | | | maternal smoking and alcohol consumption during pregnancy, and maternal energy intake during pregnancy | | | | |
| ***Schneuer et al.*** | prospective, nested case-control study | 5109 | Australia multiethnic | $1^{st}$ trim. (10-14 w) | maternal age, parity, smoking during pregnancy, maternal weight, previously diagnosed hypertension, previously diagnosed diabetes, season at sampling, country of birth, or socioeconomic disadvantage | 25(OH)D | - | Low 25(OH)D serum concentrations in the first trimester of pregnancy are not associated with adverse pregnancy outcomes and do not predict complications | |
| ***Aji et al.*** | prospective cohort | 232 | Indonesia | $1^{st}$ and $3^{rd}$ trim. | maternal age, pre-pregnancy BMI and gestational age at delivery | 25(OH)D | Deficiency: <12 ng/mL Insufficiency: 12-19 ng/mL Sufficiency ≥ 20 ng/mL | Not associated with neonatal anthropometry at birth ($p>0.05$) (LBW, birth length, low HC) | |
| ***Yuniati et al.*** | prospective cohort | 203 | Indonesia | $1^{st}$ trim. | maternal age, pre-pregnancy body mass index, and parity | 25(OH)D | deficient <20 ng/mL, insufficient 20–29.99 ng/mL and normal ≥30 ng/mL | No significant association reported between maternal serum Vit D and neonatal birth weight, before and after adjustment for confounders | |
| ***Park et al.*** | prospective | 523 | Korea | $1^{st}$ trim. | maternal age, pre-pregnancy body mass index, BMI at booking, BMI at GDM screening, gestational age at sampling, previous GDM, Vit D intake | 25(OH)D | Sufficient ≥ 50 nmol/L, borderline 25-49.9 nmol/L, insufficient <25 nmol/L | birth weight was independent of maternal serum 25-OH-D levels during the first, second and third trimester of pregnancy. | neither GDM prevalence nor fetal growth during pregnancy is associated with Vit D deficiency at 1st trim. in Korean women |

| | | | | | | | | | |
|---|---|---|---|---|---|---|---|---|---|
| | | | | | and season of sampling | | | | |
| **2nd trimester** | | | | | | | | | |
| ***Liu et al.*** | Retrospective cohort study | 15724 | China | 26-28 w | maternal age at delivery, education level, employment status, parity, history of macrosomia, pre-pregnancy BMI, height, use of folic acid supplements, maternal serum concentrations of triglycerides, high density lipoprotein cholesterol, calcium and hemoglobin in the 1st trim. | 25(OH)D | Severe deficiency: <10.0 ng/mL deficiency: 10.0-19.9 ng/mL insufficiency: 20.0-29.9 ng/mL sufficiency: >30.0 ng/mL | severely 25(OH)D deficient mothers had a decreased risk of LGA compared with those with sufficient 25(OH)D concentrations (Adjusted odds ratio (OR): 0.65; 95% confidence interval (CI): 0.43 0.96). | Compared to mothers with no 25(OH)D deficiency and no GDM, those with both 25(OH)D deficiency and GDM had 0.15 (95% CI: 0.09, 0.21) higher infant birth weight Z-score and a higher risk of LGA (OR: 1.29; 95% CI: 1.09, 1.52). Maternal 25(OH)D deficiency and GDM had additive interaction on the risk of LGA (relative risk due to interaction: 0.18). |
| ***Chen et al.*** | retrospective cohort | 2814 | China | 14-19 w | age, BMI, parity, season of blood collected | 25(OH)D | - | the hazard ratio (HR) (95% CI) per standard deviation (SD) increase of maternal serum 25(OH)D concentrations was 0.849 (95% CI 0.726, 0.993) for LBW | Higher early pregnancy Vit D was associated with a lower risk of GDM, cesarean section, preterm birth, and LBW |
| ***Casey et al.*** | Prospective HAPO study | 1585 | Northern Ireland | 24-32 w | season of sampling, maternal age at OGTT, maternal BMI at OGTT, smoker during pregnancy, alcohol use during pregnancy, family history of diabetes, | 25(OH)D | 25(OH)D quintiles (≤25 nmol/l, 25·01–49·9 nmol/l, 50–74·9 nmol/l, 75–99·9 nmol/l and ≥100 nmol/l) | birth weight standard deviation scores (SDS) and birth length SDS were significantly associated with maternal total 25(OH)D P=0.002 (95% CI 0.00-0.10) and P=0.03 (95% CI 0.01-0.13) | There were no significant associations with maternal 25(OH)D and other measures of neonatal anthropometrics or markers of neonatal glycaemia |

| | | | | | | | | | |
|---|---|---|---|---|---|---|---|---|---|
| | | | | | gestational age at delivery, sex of neonate, parity, systolic blood pressure at OGTT, maternal height, fasting plasma glucose and maternal education | | | | |
| ***Mosavat et al.*** | prospective | 890 | Australia | 2nd trim. (18 w) | Seasonal effect, maternal age, pre-pregnancy and gestational weight, race/ethnicity | 25(OH)D | < 30 nmol/L (severe Vit D deficiency), 30–49 nmol/L (mild Vit D deficiency), 50–78 nmol/L (normal VIt D level), and > 78 nmol/L (ideal Vit D level) | Maternal serum levels of 25(OH)D were positively associated with birth weight (r = 0.10, p = 0.003), body length (r = 0.10, p = 0.005) and head circumference (r = 0.10, p = 0.007) of the neonate.<br>No association with the risk for low birth weight | Low maternal serum levels of 25(OH)D are associated with GDM gestational diabetes, and race/ethnicity may modify this relationship |
| ***Morley et al.*** | prospective | 374 | Australia | <16w & at 28-32 w | infant sex, maternal height, whether first child, whether the mother smoked in pregnancy, and season when blood sample was taken | 25(OH)D | Low 25(OH)D group <28 nmol/L | Low maternal 25(OH)D in late pregnancy is associated with reduced intrauterine long bone growth [knee-heel length 4.3 mm smaller (95% CI -7.3, -1.3) & reduced mid-upper arm (0.1, 95% CI -0.06, 0.3) and slightly shorter gestation | no evident relationship between birth size measures and maternal 25(OH)D or PTH |
| ***Gernand et al.*** | prospective | 792 | USA | 12-26 w | latitude, obesity status, race, treatment group, risk group, | 25(OH)D | Tested different cutoffs<br>Deficiency <30 | Maternal Vit D status in the 2nd trim. is associated with risk of SGA | High risk for preeclampsia development study group<br>Under treatment with low doses of aspirin |

| | | | | | | | | | |
|---|---|---|---|---|---|---|---|---|---|
| | | | | | gestational age at blood sampling, season at blood sampling, height, marital status, smoking, education, age, and infant sex | | nmol/L<br>Inadequacy 30-50 nmol/L<br><br>Insufficiency <75 nmol/L | among all women, and in the subgroups of white and nonobese women<br>No association in black or obese women | |
| ***Burris et al.*** | prospective | 1067 white<br>236 black | USA | 2nd trim. | season of blood draw, maternal age, pre-pregnancy body mass index and race | 25(OH)D | severe deficiency (<25 nmol/L), deficiency (25 to <50 nmol/L), insufficiency (50 to <75 nmol/L), and sufficiency (≥75 nmol/L) | The odds of SGA were higher with maternal 25(OH)D levels <25 vs. ≥25 nmol/L (adjusted OR 3.17; 95% [CI]: 1.16, 8.63) | The increased odds of SGA among black vs. white participants decreased from an OR of 2.04(1.04, 4.04) to 1.68 (0.82, 3.46) after adjusting for 25(OH)D |
| ***Eckhardt et al.*** | prospective | 2473 | USA | <26 w | infant sex, infant age, maternal pre-pregnancy BMI, socioeconomic status, race/ethnicity, season of blood draw and infant feeding | 25(OH)D | Cut point for dichotomous categorization: 3<br>0 nmol/L<br><br>Group quartiles: 30, 30–49, 50–74 and ≥75 nmol/L | Infants with maternal 25(OH)D ≥30 nmol/L vs <30 nmol/L had z-score for length and for head circumference measures 0.13 (95% CI 0.03–0.23) and 0.20 (95% CI 0.11–0.28) units higher, respectively, across the first year of life | Low maternal Vit D status was associated with deficits at birth in infant weight and BMI that were recouped across the first year of life<br><br>associations with reduced measures of linear and skeletal growth were sustained from birth to 12 months |
| ***Tian et al.*** | nested case control study (preterm and term) | 2558 | USA | 4-29 weeks | age, education, parity, pre-pregnancy body mass index, season at blood draw, and smoking | 25(OH)D | - | Maternal serum concentrations of 25(OH)D in early and mid-pregnancy were positively associated with BW/GA among non-Hispanic Black male and female infants [beta (β) = 0.015, standard error (SE) = 0.007, P = 0.025; β = 0.018, SE = 0.006, P = 0.003, respectively] and non-Hispanic White male infants (β = 0.008, SE = 0.003, P = 0.02) | |
| ***Eggemoen et al.*** | Population based, multiethnic cohort study | 719 | Norway (multiethnic) | 15 & 28 w | maternal age, parity, education, prepregnancy BMI, season, | 25(OH)D | Consistently sufficient level (≥37nmol/l at GW 15 and 28) | significantly associated with birth weight, head circumference, | Sex-specific associations for abdominal circumference and sum of skinfolds were found (P for interaction <0·05) |

| | | | | | | | | | |
|---|---|---|---|---|---|---|---|---|---|
| | | | | | gestational age and neonate sex | | consistently deficient level (<37nmol/l at GW 15 and 28) increasing level (<37nmol/l at GW 15 and ≥37nmol/l at GW 28) decreasing level (≥37nmol/l at GW 15 and <37nmol/l at GW 28) | abdominal circumference and ponderal index (P<0·05 for all), when used as a continuous variable and categorized<br><br>After adjusting for ethnicity, 25(OH)D was no longer associated with any of the outcomes | |
| ***Wen et al.*** | nested case-control study | 1635 (545 women who delivered a macrosomic neonate& 1090 controls) | China | 2$^{nd}$ or 3$^{rd}$ trim. | maternal age, birthplace, intrapartum BMI, gestational weeks at birth, fetus gender, status of gestational diabetes, parity, sampling trimester, abnormal pregnancy history and sampling season | 25(OH)D | <25.0, 25.0–37.4, 37.5–49.9, <50.0, >75.0 nmol/L vs. 50.0–74.9 nmol/L) | Among women who delivered infant with macrosomia, 71.2% of the women had serum 25(OH)D concentrations <50.0 nmol/L compared with 61.1% of the control women (P < 0.001). For women with concentrations <50.0 nmol/L, they had a 33% increased risk of macrosomia compared with women whose 25(OH)D ranged from 50.0 to 74.9 nmol/L | The risk of macrosomia was significantly increased with the decreasing concentrations of serum 25(OH)D in a dose-dependent manner (P for trend = 0.001).<br>Maternal serum 25(OH)D <50.0 nmol/L was associated with the delivery of a macrosomic infant |
| ***Tint et al.*** | prospective | 292 | Asian | 26-28 w | maternal glycemic status | 25(OH)D | inadequate ≤75.0 nmol/L sufficient >75.0 nmol/L | Neonates of mothers with mid-gestation 25(OH)D inadequacy had a higher abdominal subcutaneous | Each 1 nmol/L increase in 25(OH)D was associated with a reduction in superficial subcutaneous adipose tissue (β=−0.14 ml; 95% CI: −0.24, −0.04 ml, P = 0.006) and deep |

| | | | | | | | | | |
|---|---|---|---|---|---|---|---|---|---|
| | | | | | | | | adipose tissue volume, especially deep subcutaneous adipose tissue (r = −0.206, P < 0.001) which is metabolically similar to visceral adipose tissue in adults | subcutaneous adipose tissue (β=−0.04 ml; 95% CI: −0.06, −0.01 ml, P = 0.006), but was not associated with internal adipose tissue |
| ***Godang et al.*** | prospective | 202 | Norway | 14-16 & 30-32 w | maternal BMI | 25(OH)D | - | Neonatal total body fat mass was positively associated with umbilical cord plasma 25(OH)D, P=0.02<br><br>Umbilical cord plasma - but not maternal - 25(OH)D was a significant predictor of neonatal total fat mass | Maternal and umbilical cord plasma 25(OH)D levels varied significantly with season<br><br>However, a strong positive association between maternal 25(OH)D and umbilical cord plasma 25(OH)D was reported β=0.42, P<0.001) |
| ***Bodnar et al.*** | nested case-control study retrospective | 412 SGA infants (n = 77 white and n = 34 black) non-SGA infants (n = 196 white and n = 105 black | USA | 22 w | Race/ethnicity, pre-pregnancy BMI, smoking status, season of sample collection, marital status, private insurance, use of periconceptional multivitamins at least once per week, any pre-pregnancy physical activity, and intensity of physical activity, socioeconomic status | 25(OH)D | deficiency <37.5 nmol/L sufficiency.>75 nmol/L | U-shaped relation between serum 25(OH)D and risk of SGA among white mothers (P<0.0001), with the lowest risk from 60 to 80 nmol/L<br><br>There was no relation between 25(OH)D and SGA risk among black mothers | Compared with serum 25(OH)D 37.5–75 nmol/L, SGA odds ratios (95% CI) for levels <37.5 and >75 nmol/L were 7.5 (1.8, 31.9) and 2.1 (1.2, 3.8), respectively<br><br>One SNP in the Vit D receptor gene among white women and 3 SNP in black women were significantly associated with SGA |
| ***Yu et al.*** | retrospective | 10,586 | China | 13-27 w | Gestational | 25(OH)D | Deficiency <50 | With the increase in | Low maternal Vit D levels were |

| | | | | | | | | | |
|---|---|---|---|---|---|---|---|---|---|
| | cohort | | | | diabetes, high blood pressure, parity, pre-pregnancy BMI, maternal age, occupation, education, gestational age at birth, fetal sex, complications, and season of blood collection | | nmol/L Insufficiency 50–75 nmol/L | 25(OH)D levels, the risk of SGA and LGA tended to increase and decrease, respectively. However, 25(OH)D concentration was not an effective predictor of SGA or LGA | not associated with SGA or LGA |
| ***Boyle et al.*** | prospective cohort | 1710 | New Zealand (multiethnic) | 15 weeks | Maternal BMI, ethnicity | 25(OH)D | - | Maternal 25(OH)D concentration was not associated with development of preeclampsia, spontaneous preterm birth or SGA infants | |
| ***Zhou et al.*** | prospective | 1953 | China | 16-20 w | maternal age, systolic/diastolic pressure, pre-pregnancy body mass index and serum calcium | 25(OH)D | Low levels ≤20 ng/ml medium levels 21-29 ng/ml high level ≥30 ng/ml | No significant associations with neonatal outcomes reported | higher prevalence of gestational diabetes and preterm delivery in women with high level of Vit D |
| **3rd trimester** | | | | | | | | | |
| ***Bärebring et al.*** | Prospective cohort study | 2052<br><br>trimester 1 2046<br><br>Trimester 3 1816<br><br>Both trimesters 1810 | Sweden | 1st and 3rd trim. | season of conception, origin, Vit D supplementation, Vit D status trajectory Tobacco use, exclusion of preeclampsia cases | 25(OH)D | deficiency 25(OH)D < 30 nmol/L | 1st trim.: No associations<br><br>3rd trim.: 25(OH)D ≥100 nmol/L was associated with lower odds of SGA (OR 0.3, p = 0.031) and LBW (OR 0.2, p = 0.046), compared to Vit D deficient mothers | Women with a ≥30 nmol/L increment in 25(OH)D from T1 to T3 had the lowest odds of SGA, LBW and preterm delivery<br><br>Lower 25(OH)D in early pregnancy was only associated with pregnancy loss |
| ***Shakeri et al.*** | Longitudinal, cross-sectional study | 88 | Iran | 3rd trim. | - | 25(OH)D | - | Neonatal mean birth weight, length, and head circumference of mothers who were on the 3rd tercile of 25(OH)D serum level was significantly higher | women in the 1st tercile had shorter infants compared to women in the 3rd tercile of Vit D serum levels (p = 0.004)<br><br>The OR confirmed that Vit D deficiency and insufficiency could predict 1.3 and 1.32 |

| | | | | | | | | | |
|---|---|---|---|---|---|---|---|---|---|
| | | | | | | | | than those in the 1st tercile (p < 0.001, p = 0.004 and p < 0.001, respectively | times lower birth weight and lower infants' length at birth, respectively<br><br>Vit D deficiency and insufficiency could predict 1.29 times infants with head circumference (HC) under 25th percentile |
| ***Wierzejska et al.*** | cross-sectional | 94 | Poland | term | Vit D and calcium consumption, gravidity, maternal age and education, pre-pregnancy BMI, weight gain during pregnancy, smoking, caffeine consumption, use of dietary supplements, professional activity during pregnancy, gestational diabetes, and sex of the neonate | 25(OH)D | Severe deficiency <10 ng/ml<br>Deficiency 20-30 ng/ml<br>Insufficiency 20-30 ng/ml<br>Optimal >30 ng/ml | No relationship between maternal and neonatal cord blood Vit D concentrations vs. neonatal weight, length, head, and chest circumference at birth was found (p > 0.05) | |
| ***Velkavrh et al.*** | prospective | 73 | Slovenian | 3rd trim. | - | 25(OH)D | deficiency <50 nmol/L<br>insufficiency 50-75 nmol/L<br>sufficiency 75-125 nmol/L | no significant association between maternal 25(OH)D and newborn's anthropometric measures at birth (weight p=0.35, length p=0.59 and head circumference p=0.47) | |
| ***Wang et al.*** | prospective | 1978 | China | At delivery | maternal age, pre-pregnancy BMI, gestational age, season of blood draw, trimester, GDM, preeclampsia, Vit D supplemental, Vit D-calcium co- | 25(OH)D | - | Maternal Vit D deficiency significantly increased the risk of neonatal low birth weight (LBW) (aOR 2.83; P = 0.005) and small-for-gestational-age | maternal Vit D deficiency independently increased the risk of gestational diabetes mellitus (GDM) (adjust OR, aOR 1.08; P = 0.026) |

| | | | | | | | | | |
|---|---|---|---|---|---|---|---|---|---|
| | | | | | supplementation, birth weight, infant gender, preterm delivery, IUGR, SGA, neonatal/maternal Vit D deficiency | | | (SGA) (aOR 1.17; P = 0.015).<br><br>Vit D supplementation during pregnancy significantly reduced the risk of giving birth to LBW infants (OR = 0.47, 95%CI = 0.33-0.68, P<0.001). | |
| ***Arora et al.*** | prospective | 200 | India | At delivery | adjusted | 25(OH)D | Deficiency <20 ng/ml Insufficiency <32 ng/ml | significant association of hypovitaminosis D with low-birth-weight neonates (p = 0.01). | consecutive deliveries (included pregnancy pathology, preterm births etc) |
| ***Kawakib et al.*** | prospective | 100 elective cesarean section | Iraq | At delivery | - | 25(OH)D3 | Deficiency <20 ng/mL Insufficiency 20-29.9 ng/mL Normal ≥ 30 ng/mL | Vit D deficiency among pregnant women can be directly linked with adverse neonatal outcomes like low Apgar score, low birth weight and head circumference, and respiratory distress syndrome (p<0.001) | |
| ***Lee et al.*** | prospective observational study | 575 | Malaysia | At delivery | maternal age and BMI | 25(OH)D | Deficiency <20 ng/mL Insufficiency 20-29 ng/mL Adequacy ≥30 ng/mL | no statistically significant association between Vit D status with maternal and neonatal outcomes (low birth weight baby and neonatal intensive care unit admission) among the three groups of Vit D status (p>0.05) | GDM prevalence was higher in pregnant women with Vit D deficiency (p = 0.024). |
| ***Laird et al.*** | prospective | 172 | Seychelles | At delivery | maternal MeHg, child's sex, alcohol and tobacco use in pregnancy, diabetes, gestational age, maternal age, | 25(OH)D | Deficiency <30 nmol/L Insufficiency 30-49.9 nmol/L Sufficiency ≥50 nmol/L | Maternal 25(OH)D concentrations were not associated with child anthropometric or neurodevelopmental outcomes | Small Vit D variability as Vit D sufficient mothers were 98% of sample |

| | | | | | | | | | |
|---|---|---|---|---|---|---|---|---|---|
| | | | | | delivery weight gain, maternal BMI at enrolment, mean DHA and mean AA concentrations, Hollingshead SES, and number of other living children | | | A higher Vit D status was not a limiting factor for neonatal growth or neurocognitive development in the first 5 years of life | |
| ***Sandal et al.*** | prospective | 45 | Turkey | At delivery | - | 25(OH)D3 | VIt D deficient group <20 ng/mL<br>Vit D sufficient group >20 ng/mL | No significant differences on neonatal anthropometric parameters at birth | The primary outcome of the study was the association of circulating sclerostin and neonatal parameters |
| ***Dullaert et al.*** | prospective cohort | 240 | Belgium | At delivery | parity, maternal age, fetal sex, gestational diabetes, maternal glucose level, and preeclampsia | 25(OH)D | Deficiency ≤20 ng/mL<br>Insufficiency 21-29 ng/mL | significant association was found between Vit D level <10 ng/mL and birthweight <10th percentile, but in multivariate analysis there was no significant contribution from low Vit D<br><br>Maternal Vit D at delivery does not correlate with birthweight percentile | |
| ***MohammadBeigi*** | descriptive cross-sectional study | 106<br><br>53 Iranians and 53 Afghan refugees' women | Iran | At delivery | Gestational age | 25(OH)D | Deficiency <10 ng/mL<br>Insufficiency 10-30 ng/mL<br>Sufficiency >30 ng/mL | Maternal and neonatal 25(OH) D levels did not influence neonatal anthropometry | there was a significant correlation between maternal serum 25(OH)D and that of their neonates (P=0.000, R=0.62) |
| **Longitudinal measurements of Vit D throughout pregnancy** | | | | | | | | | |
| ***Francis et al.*** | prospective cohort | 321 | USA | 10-14 w<br>15-26 w<br>23-31 w &<br>33-39 w | maternal age, race/ethnicity, gestational age at blood draw, education, pre-pregnancy BMI, marital status and insurance | 25(OH)D | Cutoffs <50 nmol/L,<br>50–75 nmol/L,<br>& >75 nmol/L | women with an overweight/obese BMI: 25(OH)D <50 nmol/L at 10-14 w was associated with lower birthweight z-score (0.56; 95% CI: -0.99, -0.13) and length (-1.56 cm; 95% CI: -3.07, -0.06) | women with normal BMI: 25(OH)D <50 nmol/L at 10-14 w was associated with lower sum of skinfolds (-2.64 mm; 95% CI: -5.03, -0.24), at 23-31 GW was associated with larger birthweight z-scores (0.64; 95% CI: 0.03, 1.25), and at 33-39 w with both higher birthweight z-score (1.22; 95% CI: 0.71, 1.73) |

| | | | | | | | | | |
|---|---|---|---|---|---|---|---|---|---|
| | | | | | | | | at 23-31 w was associated with shorter length (-2.77 cm; 95% CI: -13.38, -4.98) and lower sum of skinfolds (-9.18 mm; 95% CI: -13.38, -4.98) | and longer length (1.94 cm; 95% CI: 0.37, 3.52) |
| ***Boghossian et al.*** | retrospective | 252 (220 African American, 32 Caucasians) | USA (interacial) | 11-21 w<br>26-29 w<br>36 w | maternal ethnicity/race, age, body mass index, smoking statu, caloric intake, corrected serum calcium, and newborn sex | 25(OH)D | Deficiency <20 ng/ml<br>Insufficiency 20–29 ng/ml<br>Sufficiency >29 ng/ml | Newborns of deficient mothers had lower total bone mineral density (β −0.009 g/cm2; 95% CI −0.016, −0.002), while lean mass (−217 g; −391, −43) and birthweight (−308 g; −540, −76) were significantly negatively associated among males only | |
| ***Benaim et al.*** | Prospective cohort | 168 | Brazil | 5-13w<br>20-26w<br>30-36w<br>All trimesters | maternal education, early pregnancy BMI, skin colour, smoking habit, alcohol consumption and energy intake | 25(OH)D | sufficiency ≥50 nmol/L; insufficiency <50 nmol/L | Mean rate of change during pregnancy in 25(OH)D was directly associated with birthweight z-score (β=0.36, 95% CI 0.07, 0.65), LGA risk (IRR: 1.97, 95% CI 1.07; 3.63) and preterm birth (IRR: 7.35, 95% CI 2.99; 18.07). | |
| ***Chen et al.*** | Prospective, population-based cohort | 3658 | China | Any stage of pregnancy | BMI before pregnancy, maternal age and season of blood sample, maternal socioeconomic status, maternal periconceptional multivitamin use and parity | 25(OH)D | Deficiency <20 ng/mL<br>Insufficiency 20-29.9 ng/mL<br>Sufficiency ≥30 ng/mL | positive correlation between maternal serum 25(OH)D level and offspring birth weight (r=0.477, P<0.001) | After adjustment for confounders, relative risk (RR) for LBW infants was 12.31 (95% CI: 4.47, 33.89) among subjects with Vit D deficiency and 3.15 (95% CI: 1.06, 9.39) among subjects with Vit D insufficiency<br><br>RR for SGA infants was 6.47 (95%CI: 4.30, 9.75) among subjects with Vit D deficiency and 2.01 (95%CI: 1.28, 3.16) among subjects with Vit D insufficiency |
| ***Bo Chen et al.*** | prospective | 3080 (included | China | >8 w | Maternal age, gestational age, | 25(OH)D | deficiency: <50 nmol/L, | In the Vit D deficient group, the | inclusion of cases with gestational pathology |

| | | | | | | | | | |
|---|---|---|---|---|---|---|---|---|---|
| | | pathology) | | | season, education level, weekly time outdoors, Vit D supplement, calcium supplement and trace element supplement during pregnancy | | insufficiency: 50 - 75 nmol/L sufficiency: ≥75 nmol/L | occurrence of spontaneous abortion and SGA was higher than those of other groups (P=.018 & P=.016 respectively) | |
| ***Pereira-Santos et al.*** | prospective | 327 | Brazil | Up to 34w | maternal education level, time of onset of prenatal care, number of consultations, maternal BMI, gestational age, neonatal sex, maternal smoking, use of closed clothing season, socioeconomic class and maternal religion | 25(OH)D | Deficient <50 nmol/l, insufficient 50–75 nmol/l and sufficient >75 nmol/l | Vit D serum concentration had a direct and statistically significant association with birth weight<br>Each nmol increase in maternal Vit D serum concentration correlated with an increase in birth weight of 3,06 (95 % CI −1·37, 4·75) g (P ≤ 0·01) | |
| | | | | **RCTs and Systematic Reviews** | | | | | |
| ***Fang et al.*** | metanalysis | 16 studies | | | | | | maternal Vit D deficiency led to an increased risk of a low birthweight neonate (OR = 2.39, 95% CI: 1.25-4.57 | neonates of deficient mothers had a lower birthweight of only 0.08 kgr (95% CI: -0.10 to -0.06) compared to controls |
| ***Santamaria et al.*** | Systematic review & metanalysis | 30 studies 35032 mother-offspring pairs | | | | | | Low prenatal Vit D levels were associated with lower birth weight (mean difference MD -100.69; 95% CI -162.25, -39.13), increased risk of small-for-gestational-age (OR 1,55; 95% CI 1.16, | No associations were observed between prenatal Vit D status and other growth parameters at birth, age 1 year, 4-6 years or 9 years, nor with diabetes type 1<br><br>Prenatal Vit D may play a role in infant adiposity and accelerated postnatal growth |

| | | | | | | | | | |
|---|---|---|---|---|---|---|---|---|---|
| | | | | | | | | 2.07) and an elevated weight in infant at the age of 9 months (MD 119.75; 95% CI 32.97, 206.52) | |
| ***Tous et al.*** | Systematic review and metanalysis | 54 | | | | | | Vit D deficient mothers (<30 nmol/L) had neonates with lower birthweight (MD −87.82g; 95% CI −119.73, −55.91 g), head circumference (MD −0.19 cm; 95% CI −0.32, −0.06 cm) and a higher risk of small for gestational age infants (OR 1.59; 95% CI 1.24, 2.03) compared to mothers with Vit D levels ≥30 nmol/L.<br><br>Vit D insufficiency (<50 nmol/L) was associated with a higher risk of SGA and PTB (OR 1.43; 95% CI 1.08, 1.91 and OR 1.28; 95% CI 1.08, 1.52, respectively) | |
| ***De-Regil et al.*** | Cochrane metanalysis | 15 trials 2833 women | | | | | | Vit D supplemented women during pregnancy, less frequently had a baby with a birthweight below 2500 g than those receiving no intervention or placebo (RR 0.40; 95% CI 0.24 to 0.67, moderate quality) | There was also some indication that Vit D supplementation increases infant length (mean difference (MD) 0.70, 95% CI -0.02 to 1.43; four trials, 638 infants) and head circumference at birth (MD 0.43, 95% CI 0.03 to 0.83; four trials, 638 women) |
| ***Aghajafari et al.*** | Systematic review and metanalysis | 31 studies | | | | | | Maternal 25(OH)D insufficiency was associated with small for gestational age infants (1.85, 1.52 to 2.26). | Pregnant women with low serum 25(OH)D levels had an increased risk of bacterial vaginosis and low birthweight infants |
| ***Pérez-Lopéz et al.*** | Systematic review and metanalysis of RCTs | 13 trials 2299 pregnancies | | | | | | Birth weight and birth length were significantly greater for neonates in the Vit D supplemented group; mean difference: 107.6 g (95% CI 59.9-155.3 | Incidence of preeclampsia, GDM, SGA, low birth weight, preterm birth, and cesarean section were not influenced by Vit D supplementation |

| | | | | | | | | | |
|---|---|---|---|---|---|---|---|---|---|
| | | | | | | | | g) and 0.3 cm (95% CI 0.10-0.41 cm), respectively | |

*Table S2: Studies on the effects of maternal Vit D status during gestation on neonatal anthropometric indices. FGR: fetal growth restriction, SGA: small for gestational age, EFW: estimated fetal weight, AGA: appropriate for gestational age, LGA: large for gestational age, GDM: gestational diabetes mellitus, LBW: low birthweight, HC: head circumference, PTB: preterm birth, SNP: single nucleotide polymorphism*
