# Supplementary material for: The Gestational Effects of Maternal Bone Marker Molecules on Fetal Growth, Metabolism and Long-Term Metabolic Health: A Systematic Review
Source: Int J Mol Sci. 2022 Jul 28;23(15):8328. doi: 10.3390/ijms23158328 (PMC9368754; doi:10.3390/ijms23158328)
Supplement: Supplementary file 1 [file ijms-23-08328-s001.zip › Table S3_Biomarkers_Birth anthropometrics.pdf]

Author	Type of study	N	Ethnicity	Trimester	Adjusted for covariates	Maternal Marker Studied	Outcome	Other outcomes/Comments
Briana et al.	Prospective	80<br>30 IUGR<br>30 LGA<br>30 AGA	Greece	At labor	-	sclerostin	Maternal and fetal sclerostin concentrations did not differ among LGA, IUGR, and AGA groups	In LGA group, maternal concentrations were elevated in cases of GDM (b=11.861, 95% CI 0.517-23.205, p = 0.041)<br><br>Included gestational pathology cases in IUGR and LGA groups
Mastorakos et al.	Prospective	100	Greece	2nd and 3rd trim.	2nd trim. insulin levels, gestational age, and fetal sex	sclerostin	2nd trim.: positive correlation between maternal sclerostin levels and neonatal birthweight (r=0.632, P<0.001)	Maternal serum sclerostin during 2nd trim. was the best positive predictor of birthweight (Standardized Beta Coefficient=0.568, P=0.003, adjusted R2=0.264)
Godang et al.	prospective	202	Norway	30 – 32 w	-	sclerostin	No significant effects of maternal sclerostin on neonatal Bone Mineral Content were found	Other maternal bone metabolism biomarkers studied (a-klotho, fibroblast growth factor 23 and 25(OH)D), showed no significant correlations either
Wei et al.	Nested-case control study	230 with LBW neonates<br>3 controls	China	All three trim.	Maternal age and pre-pregnancy BMI	osteocalcin	mothers with elevated osteocalcin serum levels during the 1st and 2nd trim., had a 2.3- (adjusted ORs=2.29, 95% CI: 1.11–4.72, p=0.025) and 1.59 - times higher risk (ORs=1.59, 95%CI: 1.03–2.45, p=0.036) of a low birthweight neonate respectively	3rd trim.: No significant associations were found
Briana et al.	prospective	40<br>20 AGA<br>20 IUGR	Greece	At labor	-	Osteoprotegerin &<br>sRANKL	No significant differences in osteoprotegerin or sRANKL concentrations were observed between AGA and IUGR groups	
Essley et al.	prospective longitudinal study	155<br>(adolescent pregnancies)	USA	Mid-gestation and at delivery			osteoprotegerin concentrations in cord blood were correlated with maternal osteoprotegerin concentrations and	no direct correlations between maternal osteoprotegerin levels and neonatal birthweight or ponderal index were reported

							were negatively associated with infant birth weight z-score (P=0.02) and ponderal index (P=0.02)	

Table S3: Studies on the effects of maternal bone turnover markers during gestation on neonatal anthropometric indices. FGR: fetal growth restriction, IUGR: intrauterine growth restriction, SGA: small for gestational age, EFW: estimated fetal weight, AGA: appropriate for gestational age, LGA: large for gestational age, LBW: low birthweight
