# Supplementary material for: The Gestational Effects of Maternal Bone Marker Molecules on Fetal Growth, Metabolism and Long-Term Metabolic Health: A Systematic Review
Source: Int J Mol Sci. 2022 Jul 28;23(15):8328. doi: 10.3390/ijms23158328 (PMC9368754; doi:10.3390/ijms23158328)
Supplement: Supplementary file 1 [file ijms-23-08328-s001.zip › Table S4_Fetal Metabolism.pdf]

Author	Type of study	N	Ethnicity	Trimester	Adjusted for covariates	Maternal Marker Studied	Cutoffs	Outcome	Other outcomes/Comments
Casey et al.	prospective	1150	Northern Ireland, UK	28 w	season of sampling, maternal age at OGTT, maternal BMI at OGTT, smoker during pregnancy, alcohol use during pregnancy, family history of diabetes, gestational age at delivery, gender of neonate, parity, systolic blood pressure at OGTT, maternal height, and maternal education	25(OH)D	25(OH)D quintiles: ≤25nmol/L, 25.01–49.99nmol/L, 50–74.99nmol/L, 75–99.99nmol/L and ≥100nmol/L	no significant associations between maternal 25(OH)D levels and cord blood glucose levels, HOMA-IR and HOMA-β at delivery	
Godang et al.	prospective	202	Norway	14-16 & 30-32 w	Maternal BMI	25(OH)D	-	no significant associations between maternal 25(OH)D and umbilical cord plasma C-peptide levels maternal BMI (β=−0.02, P=0.84)	

Table S4: Studies on the effects of maternal bone turnover markers during gestation on fetal metabolism. OGTT: oral glucose tolerance test, HOMA-IR: homeostatic model assessment for insulin resistance
