# Supplementary material for: The Gestational Effects of Maternal Bone Marker Molecules on Fetal Growth, Metabolism and Long-Term Metabolic Health: A Systematic Review
Source: Int J Mol Sci. 2022 Jul 28;23(15):8328. doi: 10.3390/ijms23158328 (PMC9368754; doi:10.3390/ijms23158328)
Supplement: Supplementary file 1 [file ijms-23-08328-s001.zip › Table S5_Future Health.pdf]

Author	Type of study	N	Ethnicity	Trimester	Adjusted for covariates	Maternal Marker Studied	Cutoffs	Outcome	Other outcomes/Comments
Neelon et al.	prospective	476	US (multiracial)	early to mid-pregnancy<br><br>(no gestational age criterion)	maternal race/ethnicity, age, smoking during pregnancy, education, pre-pregnancy body mass index, infant sex, household income, and season of blood draw.	25(OH)D	Categorized data in quartiles	Reduced maternal 25(OH)D was associated with lower birth weight for gestational age z-scores (-0.43 units; CI -0.79, -0.07; p=0.02 for Q1 and -0.56 units; CI -0.89, -0.23; p=0.001 for Q2), but higher 1-year weight-for-length (0.78 units; 0.08, 1.54; p=0.04) and 3-year BMI z-scores in offspring (0.83 units; 0.11, 0.93; p=0.02)	
Leffelaar et al.	prospective	3730	The Netherlands Multiethnic	Early pregnancy 12-14 w	fetal sex, maternal height, parity, maternal age, smoking, maternal pre-pregnancy BMI, educational level and duration of exclusive breastfeeding	25(OH)D	Deficient: ≤29·9 nmol/l<br>insufficient: 30–49·9 nmol/l<br>adequate: ≥50 nmol/l	Neonates born to mothers with a deficient Vit D status showed accelerated growth in weight and length during the first year of life	compared with infants born to mothers with an adequate Vit D status, infants born to mothers in the deficient category had significantly higher weights at months 6 and 9<br>Analyses of length showed that infants in the deficient category were significantly smaller at month 1, but significantly larger at 12 months of age
Eckhardt et al.	Retrospective	2473	US multiethnic	<26 w	infant age, maternal socioeconomic status, maternal race/ethnicity, season of maternal blood draw, infant feeding mode	25(OH)D	four categories (<30, 30–49, 50–74 and ≥75 nmol/L).	Infants with maternal 25(OH)D ≥30 nmol/L vs <30 nmol/L had z-scores for length and head circumference z-scores 0.13 (95% CI = 0.03–0.23) and 0.20 (95% CI = 0.11–0.28) units higher, respectively, across the first year of life. Similar differences in weight z-scores and BMI z-scores at birth were resolved by 12 months of age	

					and study site, infant sex and maternal pre-pregnancy BMI				
Jiang et al.	Prospective	329	China	11-29 w	maternal age, gestational weight gain, maternal Vit D and folic acid supplementation, season of serum sampling, fetal sex, complications of pregnancy, SGA, LBW, breastfeeding duration and 1-year-old infant Vit D levels	25(OH)D	Deficiency: <30 nmol/L insufficiency 30-50 nmol/L sufficiency ≥50 nmol/L]	no significant relationships were found between maternal Vit D concentrations and anthropometric indices: weight (p=0.788), length (p=0.843) and BMI (p=0.778)) during 0 to 3 years old	maternal vitamin D status appeared to be unrelated with offspring BMI-Z trajectories in multivariable logistic regression models
Morales et al.	prospective	1498 1 year<br>1344 4 years	Spain	13-15 w	area of study, child's sex, age at assessment, maternal social class and education, maternal pre-pregnancy BMI, gestational weight gain, maternal smoking in pregnancy and parity	25(OH)D3	Deficit <20 ng/mL insufficiency 20–29.9 ng/ml reference group ⩾30 ng/ml	25(OH)D3 deficit was associated with an increased risk of overweight in offspring at age 1 year (OR=1.42, 95% CI: 1.02–1.97; P=0.039); however, the association was attenuated at age 4 years (OR=1.19, 95% CI: 0.83–1.72; P=0.341)	
Crozier et al.	prospective	977	UK	34 w	sex, age at measurement, and childhood heigh, educational attainment, smoking in pregnancy, pre-	25(OH)D	Low Vit D status: <50 nmol/L<br>50 to 75 nmol/L<br>>75 nmol/L	Lower maternal Vit D status was associated with lower neonatal fat mass at birth, but with greater fat mass at 4 and 6 years of age	

					pregnancy BMI, height, parity, social class, and IOM weight gain, category, breastfeeding duration, Vit D intake at age 3 y, and physical activity at age 3 y			
Hyde et al.	Prospective cohort study	402	Australia	<16 w<br>28-32 w	Season of sample collection, child height, sex, maternal parity and smoking status at recruitment	25(OH)D	-	Maternal Vit D status in early pregnancy, in smokers, was negatively associated with offspring fat mass percentage and positively associated with lean mass (both p < 0.05). at 11-year follow up
Krishnaveni et al.	retrospective	568	India	28-32 w	Maternal age, neonatal sex, parity, religion, gestational diabetes status, socioeconomic score, season of assessment of offspring characteristics (as a proxy for their vitamin D status)	25(OH)D	deficiency <50nmol<br><br>different cutoffs <20, <30, <40, <50, <60, and <70nmol/L	At ages 5 and 9.5 years, children born to Vit D deficient mothers had smaller arm-muscle area in comparison with children born to mothers without deficiency (P<0.05). There was no difference in grip strength between offspring of women with and without vitamin D deficiency.<br>At 9.5 years, children of Vit D deficient mothers had higher fasting insulin resistance than children of nondeficient women (P=0.04)<br><br>Intrauterine exposure to low 25(OH)D concentrations is associated with less muscle mass and higher insulin resistance in children<br><br>Included 35 gestational diabetes cases

Javaid et al.	longitudinal study	160	UK	Late pregnancy	Offspring age, childhood height, socioeconomic status, supplementation status, gestational and chronological age	25(OH)D	Vit D replete >20 µg/L, Insufficient 11–20 µg/L Deficient <11 µg/L	Reduced concentration of maternal 25(OH)D during late pregnancy was associated with reduced whole-body (r=0.21, p=0.0088) and lumbar-spine (r=0.17, p=0.03) bone-mineral content in children at age 9 years<br><br>Reduced concentration of umbilical-venous calcium also predicted reduced childhood bone mass (p=0.0286).	Maternal Vit D status during late pregnancy was not associated with birthweight (p=0·24), birth length (p=0·07), placental weight (p=0·43), abdominal circumference (p=0·10), or head circumference (p=0·51).<br><br>Maternal Vit D insufficiency is associated with reduced bone mineral accrual in the offspring during childhood<br>Vit D supplementation of pregnant women, especially during winter months, could lead to long-lasting reductions in the risk of osteoporotic fracture in their offspring
Gale et al.	Prospective	466<br>440 followed up at the age of 9 months<br><br>178 at the age of 9 years	UK	Late pregnancy	-	25(OH)D	Vit D replete: >50 nmol/l, Insufficiency: 27.5-50 nmol/l Deficiency: <27.5 nmol/l	no associations reported between maternal 25(OH)D concentrations and the child's body size or measures of the child's intelligence, psychological health or cardiovascular system at birth, 9 months and 9 years of age	
Ong et al.	Prospective	910	Singapore	26-28 w	maternal ethnicity, education, smoking during pregnancy, age,	25(OH)D	Deficiency <50nmol/l insufficiency 50–75nmol/l	no statistically significant associations between maternal Vit D status and any	low prevalence (1.6 % of the cohort) of severe maternal Vit D deficiency in study population

					pregnancy BMI, total maternal energy intake, infant birth order and infant sex		sufficiency ≥75 nmol/l	of the birth outcomes: – small for gestational age (OR 1.00; 95% CI 0.56, 1.79) and pre-term birth (OR 1.16; 95% CI 0.64, 2.11) – growth outcomes – weight-for-age z-scores, length-for-age z-scores, circumferences of the head, abdomen and mid-arm at birth or postnatally – and adiposity outcomes – BMI, and skinfold thickness (triceps, biceps and subscapular) at birth or postnatally	
Rytter et al.	Prospective cohort study	850<br><br>629 (74%) self-reported anthropometrics<br><br>410 (48%) participated in the clinical examination	Denmark	30 w	Maternal pre-pregnancy BMI, maternal education, smoking during pregnancy, maternal age, parity, season of blood collection and sex	25(OH)D	In quartiles	No association was observed between maternal 25(OH)D in week 30 of gestation and offspring cardio-metabolic risk factors<br><br>However, the analyses did suggest a possible inverse association with blood pressure in females	Maternal 25(OH)D concentration was not associated with BMI, waist circumference or the adipose tissue derived hormones leptin and adiponectin in the 20-year-old offspring independent of sex<br><br>Also, no association was found with glucose metabolism measured by fasting glucose, insulin, HOMA-IR and HbA1c

									Regarding lipid metabolism, no association was found with total and LDL cholesterol, triglyceride, and apolipoprotein B<br><br>However, a weak inverse association between 25(OH)D and HDL cholesterol was found in females
Sørensen et al.	retrospective	109	Norway	Late pregnancy	Sex, season of blood draw, maternal age, parity, gestational age, and region of residence	25(OH)D	Quartile 1: ≤54 nmol/L<br>Quartile 2: 54-69 nmol/L<br>Quartile 3: 69-89 nmol/L<br>Quartile 4: >89 nmol/L	there was a trend toward a higher risk of type 1 diabetes with lower levels of Vit D during pregnancy. The odds of type 1 diabetes were more than twofold higher for the offspring of women with the lowest levels of 25(OH)D compared with the offspring of those with levels above the upper quartile	
Brekke & Ludvigsson et al.	retrospective	16070<br><br>11081 at 1 year follow-up<br>8805 at 2.5 years follow-up	Sweden	-	familial type 1 diabetes, maternal education, maternal age, delivery mode, weight increase from birth, breast-feeding duration, introduction of cow's-milk protein, fish intake	-	-	Use of Vit D -containing supplements during pregnancy was associated with reduced diabetes-related autoimmunity at 1 year of age (adjusted odds ratio: 0.707, 95%CI: 0.520–0.962, p=0.028) but not at 2.5 years	
Fronczak et al.	retrospective	233	US	3rd trim.	HLA genotype, family history of type 1 diabetes, presence of gestational diabetes mellitus, and ethnicity	-	-	Maternal intake of vitamin D via food was significantly associated with a decreased risk of IA appearance in offspring (adjusted	Maternal intake of Vit D through food during pregnancy may have a protective effect on the appearance of islet autoimmunity in offspring

								HR=0.37; 95% CI 0.17-0.78)	
Marjamäki et al.	prospective, population-based birth cohort	3,723	Finland	-	familial diabetes, genetic risk group, sex, gestational age, maternal age, maternal education, delivery hospital, route of delivery, number of earlier deliveries and smoking during pregnancy	Vitamin D daily intake	-	The maternal intake of Vit D, either from food or from supplements, was not associated with the risk of advanced beta cell autoimmunity/type 1 diabetes in offspring (HR [95% CI] for intake of Vit D from food 1.25 [0.80–1.95], for vitamin D intake from supplements 1.05 [0.95–1.16]), or with the risk of type 1 diabetes alone (HR [95% CI] for intake of vitamin D from food 0.84 [0.41–1.72], for Vit D intake from supplements 1.09 [0.99–1.20])	
Miettinen et al.	retrospective	343	Finland	Early gestation	-	25(OH)D	deficiency <25 nmol/l insufficiency 25–50 nmol/l sufficiency 50–75 nmol/l optimal levels >75 nmol/l	No difference was found in serum 25(OH)D concentrations during first trimester of pregnancy between mothers whose children later on developed type 1 diabetes, and mothers of non-diabetic, healthy children of the same age (p=0.88)	
Santamaria et al.	Systematic review & metanalysis	30 studies<br><br>2 studies for outcomes after birth	-	-	-	25(OH)D	-	Low prenatal Vit D levels were associated with lower birth weight (MD -100.69; 95%CI -162.25, -39.13), increased risk of small-for-gestational-age (OR 1.55; 95%CI 1.16, 2.07) and an elevated weight in infant at the age of 9 months (MD 119.75; 95%CI 32.97, 206.52)	No associations were observed between prenatal vitamin D status and other growth parameters at birth, age 1 year, 4-6 years or 9 years, nor with diabetes type 1<br><br>Prenatal Vit D may play a role in infant adiposity and accelerated postnatal growth
Hornsby et al.	Randomized control trial	Arm 1: 26 newborns with maternal Vit D3	-	2nd and 3rd trim.	-	25(OH)D	insufficiency <30 ng/mL	Arm 1 in comparison to arm 2, resulted in an enhanced broad-spectrum proinflammatory cytokine response of cord blood	

		supplementation of 4400 IU/d<br><br>Arm 2: 25 newborns with maternal Vit D3 supplementation of 400 IU/d						mononuclear cells to innate and mitogenic stimuli (P=.0009), with an average 1.7- to 2.1-fold increase in levels of proinflammatory cytokines (GM-CSF, IFN-g, IL-1b, IL-6, and IL-8) across stimuli, a higher gene expression level of TLR2 (P=.02) and TLR9 (P=.02), a greater than 4-fold increase in IL-17A (P=.03) production after polyclonal T-cell stimulation, and an enhanced IL-10 response	
Zosky et al.	Retrospective, longitudinal birth cohort	24 children 6 years old<br>25 children 14 years old	Australia	16 – 20 weeks	Maternal history of asthma, parental education, maternal age, birth order, breastfed for at least first 12 months, maternal smoking/alcohol use, pubertal status, current asthma/wheeze	25(OH)D	Deficiency <50 nmol/L<br>Insufficiency 50-75 nmol/L<br>Sufficiency >75 nmol/L	No associations between maternal Vit D status and atopic status in offspring<br><br>Maternal 25(OH)D deficiency was associated with wheeze and asthma (evident only in boys) at the age of 6 years.<br><br>No similar associations in 14-year-olds	Maternal Vit D status during fetal lung developmental period may impact postnatal lung growth and increase the risk for onset of lung disease
Gale et al.	prospective	440 at 9 months of age	UK	Late pregnancy	Maternal education, season of birth	25(OH)D	Quarter 1: <30 nmol/L<br>Quarter 2: 30-50 nmol/L<br>Quarter 3: 50-75 nmol/L<br>Quarter 4: >75 nmol/L	Children of mothers with 25(OH)D levels exceeding 75 nmol/L during pregnancy had increased risk of atopic disorders and eczema (OR 3.26, 95% CI 1.15-9.29, P=0.025)	After adjusting data for season of birth, the correlation attenuated (OR 2.50, 95% CI 0.80-7.77)
Pike et al.	prospective	860	UK	34 weeks	Adjusted to maternal, paternal and	25(OH)D	-	No significant associations between maternal late-pregnancy Vit D status and either asthma or wheeze at age 6 years.	

					child characteristics			Maternal vitamin D status was not associated with transient or persistent/late wheeze<br><br>No significant association between persistent/late wheeze according to atopic status<br><br>No associations were found with skin sensitisation or lung function<br><br>No evidence that exposure to higher concentrations of 25(OH)D in maternal serum during late pregnancy increases the risk of childhood asthma, wheeze or atopy	
Morales et al.	population-based birth cohort	1st year: 1724 children (wheezing) 1693 (lower respiratory tract infection)<br><br>4-6 years: 1233 (wheezing and asthma)	Spain	Late 1st trim.	infant sex, siblings at birth, season at birth, maternal age, maternal ethnicity, maternal education level, maternal social class, maternal smoking during pregnancy, breastfeeding duration, maternal history of asthma, pre-pregnancy maternal BMI and day care attendance in the first year of life	25(OH)D	Quartile 1: <21.9 ng/mL<br><br>Q2: 21.9-29.1 ng/mL<br><br>Q3: 29.2-37 ng/mL<br><br>Q4: >37 ng/mL	A trend for an independent association between higher levels of maternal 25(OH)D levels in pregnancy was noted<br><br>Decreased odds of lower respiratory tract infections in offspring (for cohort- and season-specific quartile Q4 vs. Q1, OR=0.67, 95% CI 0.50, 0.90, test for trend, P=0.016)<br><br>No association between 25(OH)D levels and risk of wheezing at age 1 year or 4 years, or asthma at age 4-6 years	higher maternal 25(OH)D levels were independently associated with lower risk of respiratory tract infections in their children during the first year of life, but no connections were noted with childhood asthma or wheezing

Dullaert et al.	Prospective cohort	240<br><br>37 infants available for 1 year follow-up	Belgium	After delivery	Parity, maternal age, fetal sex, gestational diabetes, maternal glucose level, preeclampsia, birthweight, breastfeeding, sibling to daycare	25(OH)D	Deficiency ≤20 ng/mL Insufficiency 21-29 ng/mL	In multivariate analysis, no correlation of maternal Vit D at delivery with respiratory tract infections nor atopic dermatitis in the first year of life
Erkkola et al.	population-based birth cohort	1669 children at 5 years of age	Finland	-	sex, area of birth, duration of gestation, maternal age, maternal basic education, maternal smoking during pregnancy, number of siblings, parental asthma, parental allergic rhinitis, child's atopic eczema before the age of 6 months and pets inside the house before the age of 1 year	Vit D dietary intake	-	Maternal dietary intake of Vit D was negatively related to risk of asthma [hazard ratio (HR) 0.80, 95% CI 0.64-0.99] and allergic rhinitis [HR 0.85; 95% CI 0.75-0.97]

Table S5: Studies on the effects of maternal bone turnover markers during gestation on future metabolic and endocrine wellbeing, as well as on and immunological status of offspring.
